# A Glutamatergic Spine Model to Enable Multi-Scale Modeling of Nonlinear Calcium Dynamics

**DOI:** 10.3389/fncom.2018.00058

**Published:** 2018-07-27

**Authors:** Eric Hu, Adam Mergenthal, Clayton S. Bingham, Dong Song, Jean-Marie Bouteiller, Theodore W. Berger

**Affiliations:** Department of Biomedical Engineering, University of Southern California, Los Angeles, CA, United States

**Keywords:** multi-scale modeling, spine calcium, computational model, nonlinear dynamical systems, glutamatergic synapse

## Abstract

In synapses, calcium is required for modulating synaptic transmission, plasticity, synaptogenesis, and synaptic pruning. The regulation of calcium dynamics within neurons involves cellular mechanisms such as synaptically activated channels and pumps, calcium buffers, and calcium sequestrating organelles. Many experimental studies tend to focus on only one or a small number of these mechanisms, as technical limitations make it difficult to observe all features at once. Computational modeling enables incorporation of many of these properties together, allowing for more complete and integrated studies. However, the scale of existing detailed models is often limited to synaptic and dendritic compartments as the computational burden rapidly increases when these models are integrated in cellular or network level simulations. In this article we present a computational model of calcium dynamics at the postsynaptic spine of a CA1 pyramidal neuron, as well as a methodology that enables its implementation in multi-scale, large-scale simulations. We first present a mechanistic model that includes individually validated models of various components involved in the regulation of calcium at the spine. We validated our mechanistic model by comparing simulated calcium levels to experimental data found in the literature. We performed additional simulations with the mechanistic model to determine how the simulated calcium activity varies with respect to presynaptic-postsynaptic stimulation intervals and spine distance from the soma. We then developed an input-output (IO) model that complements the mechanistic calcium model and provide a computationally efficient representation for use in larger scale modeling studies; we show the performance of the IO model compared to the mechanistic model in terms of accuracy and speed. The models presented here help achieve two objectives. First, the mechanistic model provides a comprehensive platform to describe spine calcium dynamics based on individual contributing factors. Second, the IO model is trained on the main dynamical features of the mechanistic model and enables nonlinear spine calcium modeling on the cell and network level simulation scales. Utilizing both model representations provide a multi-level perspective on calcium dynamics, originating from the molecular interactions at spines and propagating the effects to higher levels of activity involved in network behavior.

## Introduction

The calcium ion is a key biochemical signaling molecule for cellular function, and a number of studies have demonstrated its importance in numerous cell types. Calcium is known to be involved in regulation of gene transcription factors (Bading et al., 1993; Dolmetsch et al., 1998), muscle contraction (Ebashi and Endo, 1968; Weber and Murray, 1973), bone formation (Zhu and Prince, 2012), cell metabolism (Contreras et al., 2010) and apoptosis (Mattson and Chan, 2003). Within neurons, calcium has an especially critical role in modulating communication and network activity (Zucker, 1999; Emptage et al., 2001). Calcium has been extensively investigated in postsynaptic spines due to its involvement in various signaling cascades that lead to synapse formation and plasticity, cellular mechanisms which underlie learning and memory.

Early experimental studies on spine calcium focused on measuring calcium concentration changes at postsynaptic spines of the CA1 pyramidal cell in response to presynaptic stimulations and action potential transients through fluorescence procedures (Sabatini et al., 2002). Other studies focused on identifying sources of calcium influx, which include voltage dependent calcium channels (Bloodgood and Sabatini, 2007), intracellular calcium stores (Holbro et al., 2009), and NMDA receptor channels (Bloodgood and Sabatini, 2009). Downstream calcium signaling pathways have also been investigated, where calcium microdomains and localized calcium signaling (Higley and Sabatini, 2012) can invoke signaling of the ubiquitous Calmodulin/CAMKII protein, which leads to AMPA receptor upregulation (Naoki et al., 2005; Zhabotinsky et al., 2006). These standalone studies have helped further our understanding of numerous processes that regulate calcium dynamics in spines, but more research is needed to study how such processes interact with each other and together influence synaptic transmission.

Computational models have also been successfully adapted to the study of calcium dynamics in spines and neurons (Shouval et al., 2002; Naoki et al., 2005; Bartol et al., 2015). The advantage of using computer models over experimental protocols is their inherent ability to provide a controlled environment and overcome limitations in size constraints, an issue common when studying subcellular spaces such as synaptic compartments. Calcium dynamics models vary in biophysiological detail and accuracy, ranging from simple phenomonological models that directly relate calcium concentration to synaptic plasticity (Shouval et al., 2002), to detailed and complete reconstruction of the calcium dynamics at a dendritic subsection of a hippocampal CA1 neuron using stochastic Monte Carlo simulations (Bartol et al., 2015). Typically, the degree of realism used for a model is often dependent on the scale of the study, where calcium models at the cellular or network scales have less physiological detail than molecular scale models.

**Figure 1:**
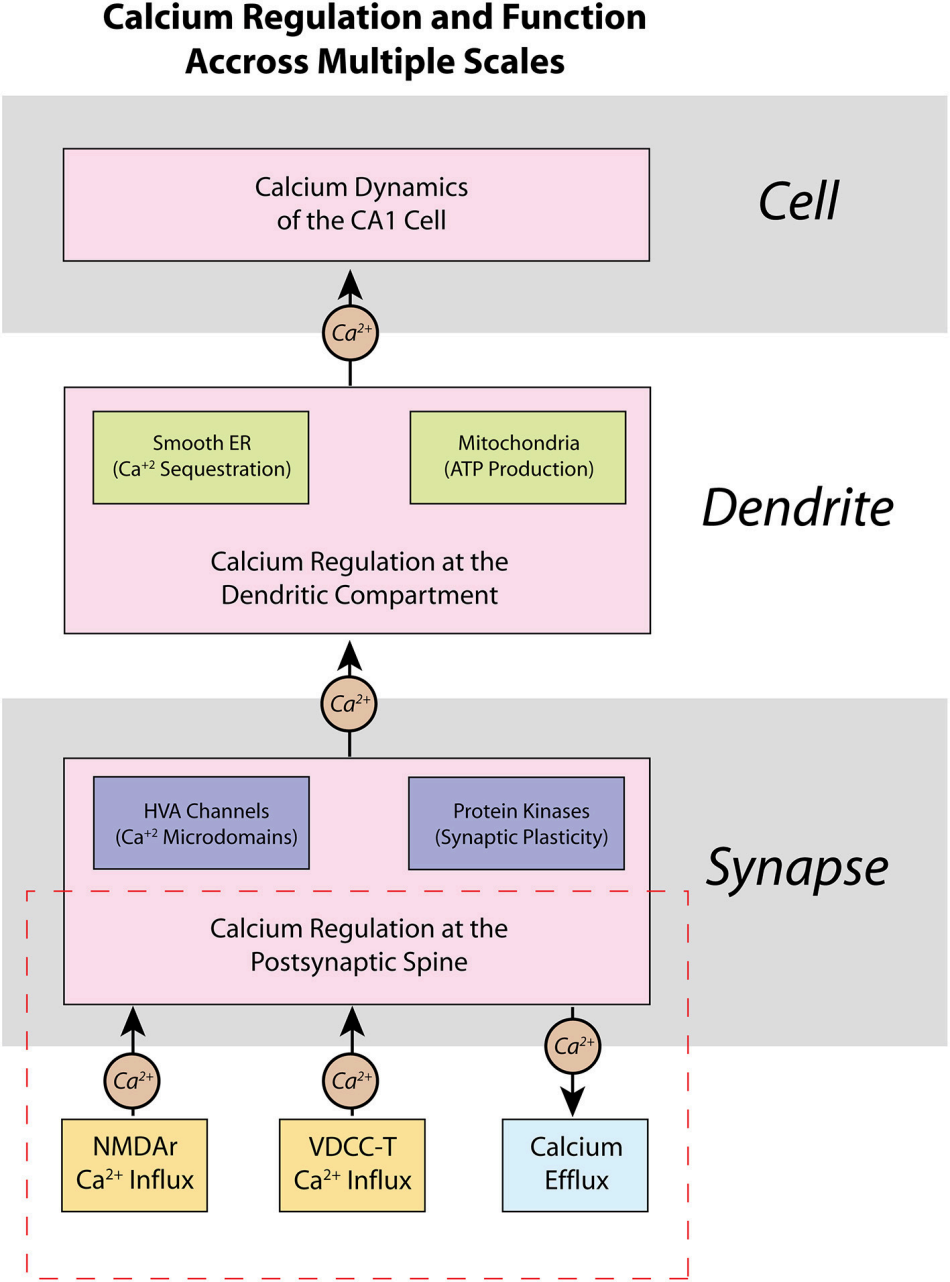
Calcium is regulated within a CA1 pyramidal cell neuron on multiple scales—from spines, to dendrites, to the entire cell itself. Presented here is a schematic representation of the regulation of calcium within a CA1 pyramidal cell neuron, as well as some of its functional properties. At the molecular level, calcium dynamics at the postsynaptic spine are controlled by various ion channels and pumps that respond to synaptic activity. Opening of high-voltage activated (HVA) calcium channels can influence other key processes such as localization of calcium microdomains, which can lead to functional changes brought about by calcium-dependent protein kinases such as activation of secondary messenger pathways and synaptic plasticity. Calcium dynamics are also important in dendrites, where many intracellular calcium stores, such as the smooth endoplasmic reticulum (ER) and mitochondria, are located. The presence of calcium stores allows calcium to influence local regulation of factors such as gene transcription and ATP production and regulation. The various sub-cellular calcium dynamics integrated together make up the changes in cell and network activity of the neuron. Our current work in this manuscript is highlighted in the dashed red rectangle in the presented hierarchy: we focus on the development of a model of calcium at the postsynaptic spine, and how our model may be applied in future work to higher hierarchies.

Scientific computation has been trending toward multi-scale modeling, where models are developed to explore biological phenomena across multiple length or hierarchical scales (Yu and Bagheri, 2016; Seo and Jun, 2017). Neural computation spans molecular (Naoki et al., 2005; Bartol et al., 2015), cellular (Jarsky et al., 2005; Migliore and Migliore, 2012), network (Hendrickson et al., 2015), and cortical systems (Markram, 2006) hierarchical scales. Thus, multi-scale modeling is especially valuable for studying calcium dynamics in neurons, because calcium induced molecular signaling cascades can have dramatic effects on patterns of activity at the cellular/network level. Calcium activity in a CA1 pyramidal neuron is not distributed equally as evidenced in experimental studies (Higley and Sabatini, 2008). Rather, calcium processes can be categorized based on location and degree of influence (Figure 1). For example, the high-voltage activated (HVA) calcium channels present on postsynaptic spines can create localized calcium microdomains—brief, high concentrations of calcium in a small area—which can then lead to protein kinase activation and induce secondary messenger cascades, eventually resulting in synaptic plasticity and larger scale changes in cell properties and network activity (Higley and Sabatini, 2012). Meanwhile, intracellular calcium stores, such as the smooth endoplasmic reticulum (ER) and mitochondria, in dendritic compartments can be involved in more metabolic processes such as gene transcription and ATP production (Li et al., 2004). If possible, use of detailed, biologically accurate computational models in large-scale simulations would add the benefit of monitoring such significant molecular-level influences in large network interaction; however, simulating such a large number of complex mechanisms and interactions in a realistic model requires a prohibitively high computational cost. Unfortunately, this computational burden limits the capability of current models to elucidate critical calcium-dependent mechanisms associated with plasticity, learning, and memory, which emerge from network level activity.

To adequately model calcium-influenced cellular and network level behaviors, it is critical to construct a model that can efficiently and accurately replicate nonlinear calcium dynamics based on the numerous calcium processes on multiple levels, starting at the spine.

The focus of this article is to present a model describing the calcium dynamics at the postsynaptic spine of a CA1 pyramidal cell, as well as a methodology to adapt the previously defined model for multi-scale simulations. The calcium model presented aims to (1) consider the variety of subcellular processes that influence calcium at the spine, and (2) enable multi-scale simulations that include the influence of said subcellular processes on calcium dynamics on a larger scale. In (1), we develop a mechanistic model that consists of various kinetic state models of receptor channels and pumps pertaining to calcium regulation at the spine; the mechanistic model is validated with experimental data from the literature and is used to study the subcellular processes involved in spine calcium dynamics. In (2), we implement a “input-output” model using the Volterra functional series trained on the nonlinear calcium dynamics from the mechanistic model; the Volterra functional series is a nonlinear systems filter that has been adapted previously to successfully model dynamics of nonlinear systems with reduced computational cost (Marmarelis and Marmarelis, 1978; Bharathy et al., 2008; Song et al., 2009a,b; Berger et al., 2010; Tu et al., 2012; Hu et al., 2015). We demonstrate that the IO model requires less time to simulate than the mechanistic model, while still reproducing the complex nonlinear dynamics that arise from calcium interactions at the spine. Thus, we propose that the IO model is better suited for multi-scale modeling of calcium. In future studies, this will allow us to investigate the effects of how the various subcellular mechanisms in which affect spine calcium dynamics can influence cell to cell interactions at the cellular and network levels.

## Materials and methods

**Figure 2:**
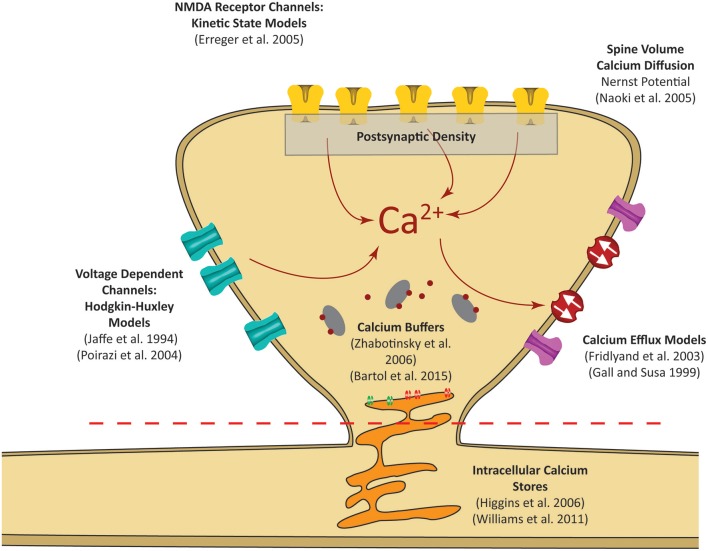
Postsynaptic calcium can be influenced by a number of factors. Our focus is on calcium dynamics at the postsynaptic spine, as was highlighted in the red dashed rectangle in Figure 1. Models for receptor channels and pumps that contribute to calcium dynamics have been developed in several studies. In our model, these sources are integrated into a single platform to construct a mechanistic model of postsynaptic calcium dynamics.

Here we first describe the models and parameters used for the mechanistic model; an overview diagram is presented in Figure 2, and a summary of the parameters is provided in Table 1. A precursor of the mechanistic model had been described previously in a conference publication (Hu et al., 2016), which was at the time an incomplete; this current model now describes the model in full with additional mechanisms, optimized parameters, and validation that is described in the results section. The development of the Input-Output model is described afterwards.

**Table 1:** List of the parameters, specifications, and models used in the mechanistic model and their sources.

**Parameter name**	**Value**	**References**
**SPINE PROPERTIES**		
Spine volume (*V*_*spine*_)	0.1 μm^3^	Stewart et al., 2005
PSD volume (*V*_*PSD*_)	0.0032 μm^3^	Stewart et al., 2005
Spine surface area	0.671 μm^2^	Stewart et al., 2005
PSD surface area	0.132 μm^2^	Stewart et al., 2005
Diffusion equation within spine	Equation 1	Naoki et al., 2005
Backpropagation attenuation	~30%	Golding et al., 2001
Nernst potential of [Ca^2+^]_i_ at 0.05 μM	−60 mV	Calculated
Nernst potential of [Ca^2+^]_i_ at 10 μM	−30 mV	Calculated
**NMDA MODEL**		
Kinetic states model	Supplementary Figure 1	Erreger et al., 2005
Number of NMDA receptors (*n*_*NMDA*_)	20	Racca et al., 2000
Percent of Ca Ion in NMDAr current	11%	Burnashev et al., 1995
Simulated NMDAr response amplitude	1.2 μM	Higley and Sabatini, 2012
**CALCIUM EFFLUX**		
PMCA Hill equation model (I)	Equation 10	Fridlyand et al., 2003
P_mCap_ (max PMCA extrusion)	0.25 pA	Calibrated
Half max concentration	0.1 μM	Fridlyand et al., 2003
NCX Hill equation with Na/Ca gradient	Equation 11	Gall and Susa, 1999
g_NaCa_ (max conductance)	0.0117 pS	Gall and Susa, 1999
Half max concentration	1.5 μM	Gall and Susa, 1999
[Ca^2+^]_i_, [Ca^2+^]_o_, [Na^+^]_i_, [Na^+^]_o_	0.05 μM, 2 mM, 10 mM, 140 mM	Gall and Susa, 1999
**VDCC MODEL**		
Predominant type	T-type channels	Higley and Sabatini, 2012
Total number of VDCCs per spine	1–20	Sabatini and Svoboda, 2000
Number of VDCCs opened during BPAP	5	Sabatini and Svoboda, 2000
T-Type single channel conductance (*g*_*VDCC*_)	7.5 pS	Perez-Reyes et al., 1998
Max [Ca^2+^] during BPAP	~600 nM	Sabatini et al., 2002
Decay of [Ca^2+^] during BPAP transient	~30 ms	Sabatini et al., 2002
**CALCIUM BUFFERS**		
Percent of calcium buffered	95%	Sabatini et al., 2002
Calmodulin (CaM)	Supplementary Figure 2	Zhabotinsky et al., 2006
CaM concentration	0.01 mM	Zhabotinsky et al., 2006
CaM Hill coefficient (*h*_*c*_)	3	Zhabotinsky et al., 2006
CaM forward rate *k*_*forward*_ ()	10e7/mM^3^ * ms	Zhabotinsky et al., 2006
CaM reverse rate (*k*_*reverse*_)	10/ms	Zhabotinsky et al., 2006
Calbindin	Supplementary Figure 2	Bartol et al., 2015
Calcium binding proteins (CBP)	Supplementary Figure 2	Naoki et al., 2005; Bartol et al., 2015
CBP concentration	0.8 mM	Calibrated
CBP forward rate (*k*_*forward*_)	247/mM * ms	Calibrated
CBP reverse rate (*k*_*reverse*_)	4/ms	Calibrated

### Mechanistic model

**Figure 3:**
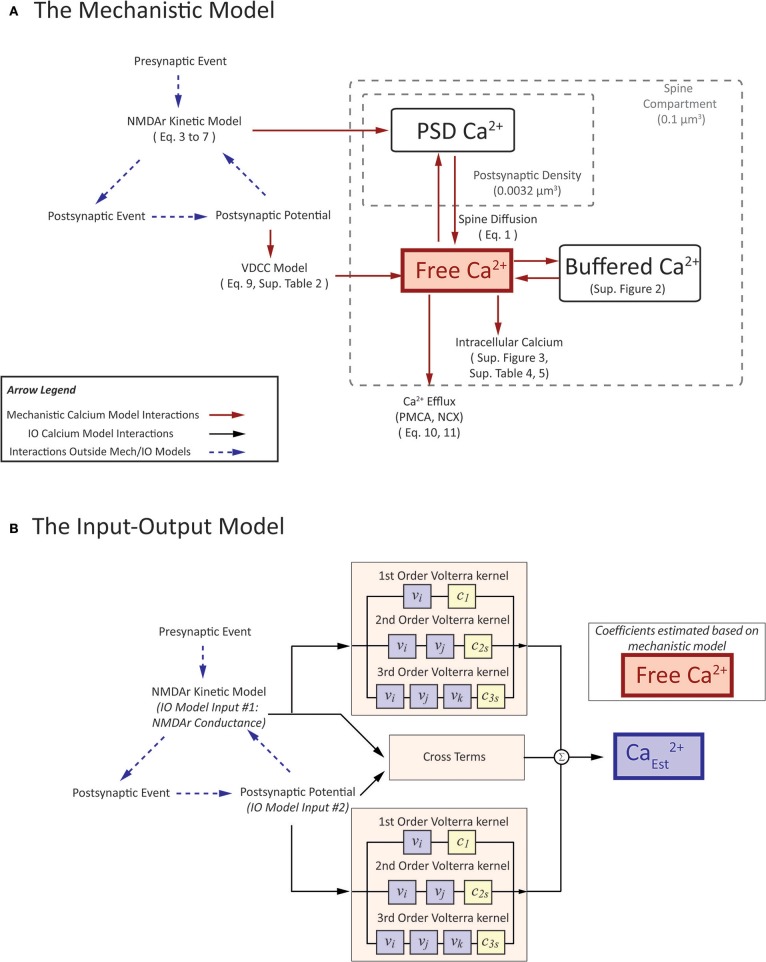
A visual depiction of **(A)** mechanistic calcium model and **(B)** the Input-Output spine calcium model. **(A)** portrays an interaction diagram of the mechanistic calcium model presented in the manuscript, with references the equations and kinetic schemas which govern the dynamics of each of the individual components. **(B)** In the IO model, the inputs and output correspond to parameters in the mechanistic model. Inputs to the IO model are NMDAr conductance and postsynaptic potential. Output is the estimate on calcium concentration in the spine. Coefficients are estimated based on the spine calcium mechanistic model, and presented in Supplementary Table 6.

The premise of the mechanistic model is to build a physiological representation of the postsynaptic spine taking into account the components that are known to significantly influence spine calcium dynamics. These components are identified based on a number of experiments, reviews, and models found in the literature (Sabatini et al., 2002; Bloodgood and Sabatini, 2007; Higley and Sabatini, 2012; Bartol et al., 2015). In the mechanistic model, influx and efflux components such as the calcium channels (NMDAr, VDCC) and calcium pumps (PMCA, NCX) are represented as calcium current sources which can add or remove calcium in the spine compartment. As calcium ions flow in and out of the spine, the concentration is determined by calculating the change in calcium divided by spine volume—the standard definition of concentration. Buffers and intracellular calcium stores interact directly with the calcium within the spine using reaction rate equations. A schematic diagram of the interactions between the components in the model is presented in Figure 3A.

Besides modeling calcium in itself, each synapse also provides postsynaptic current to the model neuron. The postsynaptic current influences the postsynaptic potential, which later influences spine calcium via voltage-dependent calcium mechanisms. The postsynaptic current is governed by both AMPA receptor channel and NMDA receptor channel kinetic rate models. The NMDA receptor channel kinetic rate model is the same model described in this Supplementary Figure 1. The AMPA receptor channel rate model is the model described in Robert and Howe (2003). These components are part of the synaptic platform which has been developed in our lab and further details can be seen in Bouteiller et al. (2011), Allam et al. (2015), and Hu et al. (2015).

#### Spine volume and diffusion

The concentration of any constituent depends on the volume of its compartment. Spines come in a variety of shapes and sizes, with different forms such as thin spines, stubby spines, and mushroom spines (Stewart et al., 2005). The postsynaptic calcium model presented here considers the composition of an average mushroom spine of a CA1 neuron, with a spine volume of 0.1 μm^3^ and the postsynaptic density volume set to 0.0032 μm^3^ (Stewart et al., 2005). Here, we model the postsynaptic density as separate compartment due to the rapid increase in local calcium concentration during ion channel activation; the calcium concentration then diffuses into the rest of the spine. Diffusion from the postsynaptic density (PSD) to the spine compartment was approximated using the spine diffusion model described by Naoki et al. (2005). The diffusion rate is modeled as:

(1) $$\begin{array}{l} {\left(\frac{d\left[Ca^{2+}\right]}{dt}\right)}_{diffusion}=D\frac{A}{d^{*}V_{spine}}\left({\left[Ca^{2+}\right]}_{PSD}-\left[Ca^{2+}\right]\right) \end{array}$$

(2) $$\begin{array}{l} \frac{d{\left[Ca^{2+}\right]}_{PSD}}{dt}=-\frac{I_{NMDA}}{2FV_{PSD}}-\;D\frac{A}{d^{*}V_{PSD}}\left({\left[Ca^{2+}\right]}_{PSD}-\left[Ca^{2+}\right]\right) \end{array}$$

where *A* is the surface area between the PSD and the rest of the spine volume, *d* is the distance between the midpoints of the PSD and the spine, *V*_*spine*_ is the total volume of the spine, and ${\left[Ca^{2+}\right]}_{PSD}$ and ${\left[Ca^{2+}\right]}_{Spine}$ is the calcium concentration at the postsynaptic density and the spine, respectively. The postsynaptic density is dependent on *I*_*NMDA*_, which is the calcium influx current from NMDA receptors. The equation for *I*_*NMDA*_ is described in Equation (3). The influx depends on Faraday's constant *F* and the volume of the postsynaptic density *V*_*PSD*_.

In our model, we consider the spine to be an isolated compartment from the rest of the cell, where the calcium does not flow out of the spine into the dendrite or vice versa. We make this assumption based on experimental evidence that spines are isolated electrically (Grunditz et al., 2008), and that less than 0.01% of the total calcium flux into the spine comes from the adjacent dendrite (Sabatini et al., 2002). As such, the model described here assumes that calcium exchange with the dendrite is insignificant and calcium dynamics from our spine model does not influence dendritic potential in our neuron model. We are aware that while dendritic calcium can be influenced by spine calcium dynamics, but in the current study, the focus of our model considers only the spine calcium and not dendrite calcium dynamics, as highlighted specifically as the red area in Figure 1. Future studies are planned that will expand our model beyond the spine level and integrate calcium across more hierarchies that had been presented in Figure 1. In the meanwhile, dendritic potential can influence spine calcium influx dynamics, as described in the following section.

#### Calcium influx

Here, we first describe the models for NMDAr and VDCC, then explain protocols to simulate calcium influx mechanisms and activate the aformentioned models.

The calcium current contribution of the NMDA channel is calculated as:

(3) $$\begin{array}{l} I_{NMDA}=g_{totalNMDA}\times V_{Ca} \end{array}$$

(4) $$\begin{array}{l} g_{totalNMDA}=\;g_{NMDA}\times n_{NMDA} \end{array}$$

*I*_*NMDA*_ indicates total calcium current that flows into the spine, *g*_*NMDA*_ is the NMDA conductance to be described in the following paragraph and *n*_*NMDA*_ represents the number of NMDA receptors with the given PSD volume and is set to 20 (Racca et al., 2000). *V*_*Ca*_ represents the calcium potential difference between intracellular and extracellular calcium, which again will be described in more detail later in the text.

The NMDA receptor channel is represented as a kinetic states model as described in Erreger et al. (2005). The model consists of 8 states that represent the resting, activation, opening, and desensitization states of the NMDA receptor channel. The magnesium ion blockade of the NMDAr channel also must be considered. To do so, the magnesium ion binding properties in the channel pore are described by:

(5) $$\begin{array}{l} g_{0}=g_{1}+\frac{g_{2}-g_{1}}{1+e^{\alpha V}} \end{array}$$

(6) $$\begin{array}{l} g_{\max}=\frac{g_{0}}{1+\left(\frac{{\left[Mg^{2+}\right]}_{o}}{K_{0}}\right)e^{-\delta zFV/RT}} \end{array}$$

(7) $$\begin{array}{l} g_{NMDA}=g_{\max}\times O\left(t\right) \end{array}$$

Where *g*_0_ represents the total conductance in the absence of any magnesium, *g*_1_ and *g*_2_ represent the open state conductances with one glutamate bound and two glutamate molecules bound, respectively. *g*_1_is set at 40 pS while *g*_2_ is set at 247 pS. The value α = 0.01 represents the steepness of the transition between *g*_1_and *g*_2_. ${\left[Mg^{2+}\right]}_{o}\;$represents the external magnesium concentration and is set at 1 mM. *K*_0_ is the equilibrium constant for magnesium set at 3.57, *F* is Faraday's Constant (9.64867.104 C mol^−1^), *R* is the molecular gas constant (8.31434 J mol^−1^ K^−1^), *z* is the valency of the calcium ion (2), and *T* is the temperature at 299.5 K. *V* represents the membrane potential. δ is the affinity between NMDAr and magnesium, which is dependent on the postsynaptic potential of the synapse; the value is set to 0.8. The variable *O*(*t*) is the open state probability governed by the kinetic rate equations for the NMDA model. The kinetic schema of the NMDAr model is presented in Supplementary Figure 1 where *O*(*t*) is represented as “Open.” The rate constants which govern NMDA kinetics are presented in Supplementary Table 1. In the kinetic model, presynaptic action potentials cause vesicle glutamate release. Thus, in our model, a presynaptic event correlates to a glutamate spike in the NMDAr kinetic states model, moving the NMDAr channel kinetics away from resting state and causing them to open. For a more extensive description of the kinetic NMDAr model, please refer to Erreger et al. (2005).

NMDAr is differentially permeable to different ions when activated. Burnashev et al. (1995) reported that on average, calcium constitutes about 11% of the total ion current when NMDAr channels are opened. However, considering NMDA current alone lead to an incorrect representation of NMDAr mediated calcium influx, since the reversal potential of calcium (+ 50 mV) is considerably different from the reversal potential of NMDAr conductance (+0 mV). Therefore, in this model we calculate the influx using the Nernst equation, which instead depends on the difference between intracellular and extracellular calcium concentration. The Nernst equation used is as follows:

(8) $$\begin{array}{l} V_{Ca}=\;-\frac{RT}{2F}\log\left(\frac{{\left[Ca^{2+}\right]}_{o}}{\left[Ca^{2+}\right]}\right) \end{array}$$

*V*_*Ca*_ represents the potential difference for calcium. *R, T*, and *F* represent the molecular gas constant, temperature, and Faraday's constant with the same values mentioned previously. ${\left[Ca^{2+}\right]}_{o}$ is the extracellular calcium set as a constant concentration of 2 mM, while [*Ca*^2+^] represents intracellular calcium concentration at the spine. Here, we assume the calcium at the spine dominates the driving force represented by the Nernst potential. It is also possible to use the calcium concentration at the postsynaptic density to calculate the potential, but due to its small volume, minor fluctuations in current can result in drastic changes in the concentration value, which would lead to more erratic changes in the potential. Therefore, we consider the use of the overall spine concentration to be a more adequate representation of the Nernst potential for calcium.

The Nernst potential is typically used to determine the potential when the spine is at during the resting state. However, for our purposes we use the Nernst potential as an estimate of the driving force for calcium influx. Our justification is thus: In our spine model, we assume that all ions besides calcium remain constant and the electrochemical force for all other ions is zero. As mentioned earlier, considering only a percentage of synaptic current influx as calcium is inaccurate, since the reversal potential between calcium is much higher than the reversal potential of synaptic current. This case is true even for the Goldman–Hodgkin–Katz flux equation, since it depends on the membrane potential—if the membrane potential moves from negative to positive, the flux is also reversed. On the other hand, using a constant value for calcium potential, i.e., when calcium is at rest at 50 nM, does not account for changes in flux induced by increased calcium levels in the spine. Therefore, the use of the Nernst potential is an estimation of the electrochemical gradient when calcium concentration is changed. The concentration of intracellular calcium is orders of magnitude lower than the concentration of extracellular calcium, so the change is not large, but still significant enough such that we believe it should be accounted for, i.e., the value of *V*_*Ca*_ when intracellular calcium is 50 nM is approximately −60 mV, but can reach −30 mV when intracellular calcium reaches at 10 μM.

Voltage dependent calcium channels (VDCCs) let calcium into the spine when there is a significant difference in membrane potential, such as action potential backpropagation from the postsynaptic neuron (Higley and Sabatini, 2012). Various types of VDCCs exist—each having different channel properties, mechanics, and functional roles—and are found on different types of cells (Catterall, 2011). For CA1 pyramidal cells, experimental evidence suggests that T-type VDCCs contribute the most to overall calcium concentration within dendritic spines (Bloodgood and Sabatini, 2007). It should be noted that L type and R type VDCCs are also present. However, the calcium influx contribution of L-types and R-types to the overall calcium concentration within spines was found to be insignificant. Instead, these channels tend to be concentrated into microdomains and activate secondary messenger pathways (Higley and Sabatini, 2012). Consequently, we consider the VDCC influx through T-type channels only.

The more specific details of the VDCC model are described in Supplementary Table 2. In general, the calcium contribution of the VDCC channel is calculated as:

(9) $$\begin{array}{l} I_{VDCC}=g_{VDCC}\cdot m^{2}\cdot h\cdot f_{drive} \end{array}$$

Where *I*_*VDCC*_ is the calcium current from the voltage dependent calcium channel. *m* and *h* are part of the Hodgkin-Huxley equation, with parameters as defined from Jaffe et al. (1994). *f*_*drive*_ is the driving force of the internal and external calcium dynamics, considered through modifications to the Hodgkin Huxley equation as reported by Poirazi et al. (2003) (equivalent to *dvf* in their model). Once again, the equations and parameters used in the model related to VDCC are presented in Supplementary Table 2. *g*_*VDCC*_ is the he single channel conductance for VDCC is set to be 7.5 pS (Perez-Reyes et al., 1998) and the average number of VDCCs opened for each AP-evoked transient is 5 (Sabatini and Svoboda, 2000).

#### Calcium efflux

Experimental evidence indicates that calcium is removed from the intracellular space through pumps and exchangers, but mechanistic details concerning calcium efflux at the postsynaptic spine are not yet fully understood. Generally, Plasma Membrane Calcium Pumps (PMCA) and Sodium-Calcium Exchangers (NCX) are the two prominent elements that participate in calcium extrusion in spines and small dendrites of CA1 pyramidal cell neurons (Scheuss et al., 2006). Overall it is thought that the constant active pumping by PMCA helps maintain the standard basal levels of calcium at ~50 nM (Carafoli, 1991), while NCX helps to quickly extrude calcium in a short amount of time, such as during an action potential (Carafoli et al., 2001). One source in the literature suggests the PMCA isoform is type PMCA2w, although details on the dynamics and extrusion rates of the isoform are lacking (Burette et al., 2010). Details on NCX at spines are even less studied, with only one source indicating that NCX is present in larger numbers in dendritic shafts than in spines, though exact numbers are unknown (Lörincz et al., 2007).

Previously published models of spine calcium use calcium extrusion models as a calibration factor to fit simulations to experimental results (Naoki et al., 2005; Bartol et al., 2015). For our model, we are interested in using more physiologically accurate representations of extrusion, but such models specifically relating to the extrusion pumps and channels in spines are currently absent from the literature. Therefore, we have decided to use extrusion models from models of other physiological systems (Gall and Susa, 1999; Fridlyand et al., 2003), and adjust parameters according to the geometry of the synapse based on the surface area density of the models presented within these papers. The adjusted parameters are detailed in the following paragraph.

Both PMCA and NCX are represented through the Hill equation, which is typically used to describe binding properties of a ligand to the receptor:

(10) $$\begin{array}{l} I=I_{MAX}\frac{{\left[Ca^{2+}\right]}^{h_{c}}}{K^{h_{c}}+{\left[Ca^{2+}\right]}^{h_{c}}} \end{array}$$

Here, *I*_*MAX*_ is the maximum calcium current extruded from the model, *K* is the equilibrium constant and *h*_*c*_ is the hill coefficient. For PMCA model parameters, the Hill coefficient is set to 2 and an equilibrium constant of 1 μM (Fridlyand et al., 2003). The maximum calcium current was optimized through gradient descent based on experimental protocols highlighted in the results section; the optimized maximal PMCA current was set to be 0.25 pA. The NCX model has a Hill coefficient of 5, equilibrium constant value of 1.5 μM and a conductance of 0.0117 pS (Gall and Susa, 1999); maximal current is then calculated from the sodium/calcium gradient and the given conductance. The higher Hill coefficient represented in the NCX model indicates a higher affinity for calcium ions when more calcium is bound. A table describing the parameter values is presented in Table 2.

**Table 2:** List of calcium efflux parameters.

**Parameter**	**PMCA model**	**NCX model**
*h*_*c*_	2	5
***K***	1 μM	1.5 μM
*I*_*MAX*_	0.25 pA	*V*_Na,Ca_^*^ 0.117 pS

Additionally, the NCX model accounts for the sodium/calcium gradient in which three sodium ions are exchanged for one calcium ion (Fridlyand et al., 2003):

(11) $$\begin{array}{l} V_{Na,Ca}=\frac{RT}{F}\left(3\text{ln}\left(\frac{{\left[Na^{+}\right]}_{o}}{{\left[Na^{+}\right]}_{i}}\right)-\ln\left(\frac{{\left[Ca^{2+}\right]}_{o}}{{\left[Ca^{2+}\right]}_{i}}\right)\right) \end{array}$$

*R, T*, and *F* represent the molecular gas constant, temperature, and Faraday's constant with the same values mentioned previously. *I*_*MAX*_ for NCX is determined based on the gradient difference. In the current model, intracellular and extracellular sodium concentrations are considered constant at 10 and 140 mM respectively. Similar to *V*_*Ca*_ from the Nernst equation, *V*_*Na,Ca*_ is an estimate of the driving force to consider how calcium efflux will change depending on calcium concentration, providing a slightly better estimate compared to keeping *V*_*Na,Ca*_ constant.

#### Buffers and intracellular calcium stores

Buffering is an integral part of calcium dynamics at the postsynaptic spine, as up to 95% of the total intracellular calcium is bound to buffers (Sabatini et al., 2002). There are numerous types of buffers that can bind calcium; in our model, we specify two types of buffers, calmodulin and calbindin, while the other possible buffers are represented as a collection of generic calcium binding proteins (CBP). In our platform, the buffer models directly influence the free calcium concentration in the spine using reaction rate equations. The kinetic schemas and descriptions of the parameters and equations are presented in Supplementary Figure 2, Table 1, and Supplementary Table 3. Calmodulin is a ubiquitous calcium buffer which plays a role in AMPA receptor upregulation and synaptic potentiation when found in the postsynaptic spine. The calmodulin buffering parameters are defined in accordance to Zhabotinsky et al. (2006). Calbindin is a binding protein with four calcium binding sites; here it is defined as a 9 states kinetic model, with parameters defined in the calcium model by Bartol et al. (2015). The CBP were calibrated after the previous two buffers were implemented, where the total buffered calcium at steady state reaches approximately 95% in the presence of all three buffers.

Intracellular calcium stores are known to play a large role in calcium dynamics, but current evidence on its impact particularly on dendritic spines in CA1 neurons remains controversial. So far it is found that approximately 19% of the total spine count contain endoplasmic reticulum (ER), with a majority of the ER-containing spines having a larger volume than others (approx. 0.06 μm^3^) (Holbro et al., 2009). The ER apparatus within the spine was shown to have no IP3 receptors present while retaining a number of ryanodine receptors (Paula-Lima et al., 2014). As such, we have included in our model state representations for SERCA pumps and ryanodine receptors, but omit IP3 receptors. Just like the buffer models, the models pertaining to the intracellular calcium stores directly interact with the free calcium concentration in the spine. We describe the kinetic schema, parameters, and equations of SERCA and ryanodine receptors in Supplementary Figure 3, Supplementary Tables 4, 5. The SERCA pump is a 2 states model with equations and parameters derived from Higgins et al. (2006). We also included the ryanodine receptors model proposed by Williams et al. (2011).

#### Inputs into the mechanistic spine calcium model

In Figure 3A, we provide a diagram of the interactions in the mechanistic model and describe the components which can influence calcium activity. Input stimulation predominantly occurs in two ways: (1) through synaptically activated transients, where presynaptic release of neurotransmitter activates the NMDA receptor channels on the postsynaptic density; and (2) AP-evoked transients, where stimulation of the postsynaptic neuron triggers an action potential, which is then backpropagated to the spines. Simulation of (1) is represented through presynaptic event-based activation of the NMDA receptor model, where a single event triggers the opening kinetics of the NMDAr model. The protocol for (2) is slightly more complex: in order to simulate AP-evoked transients in our model, we stimulate a number of synapses on the neuron model to invoke an action potential. Calcium concentration can then be measured on a stimulated or non-stimulated synapse, where the back-propagating action potential opens VDCCs and prompts calcium entry into the spine.

### Input-output model

The development of the Input-Output (IO) model for postsynaptic calcium dynamics follows a protocol similar to the IO models that had been covered in Berger et al. (2010) and Hu et al. (2015). To describe briefly, the IO model uses the Volterra functional power series, with Laguerre functions as the basis equations of the Volterra series. The single input, single output (SISO) model of the Volterra functional series and the Laguerre equations have been previously described in Hu et al. (2015). In brief, we propose to use a simplified functional representation of the system under consideration.

In the current work, a multi-input, single output (MISO) model was developed with the inputs being the two major sources of calcium influx for the calcium model: (1) postsynaptic potential (*V*), and (2) NMDAr channel conductance (*g*_*totalNMDA*_) based on glutamate-based calcium influx. One notable difference is that we herein consider the inputs to be continuous. Although we use spike trains as input to our synapse platform, the conductance and potential which are calculated from the spike trains are continuous. The use of continuous inputs contrasts with IO models that have been published previously assumed the inputs to be of binary nature (Hu et al., 2015). A pictorial representation of the mechanistic model and IO model is shown in Figure 3B, to highlight which parameters the two models have in common.

The inputs of the IO model were chosen because they represent outside activity that drives changes in calcium dynamics. The first input, the postsynaptic potential, is a factor known to drive calcium influx due to its effects on voltage dependent mechanisms (NMDAr associated channel and calcium channels). In both mechanistic and IO models, the postsynaptic potential depends on the neuron cell model. In our simulations, the Izhikevich model (Izhikevich, 2003) is used for most simulations except simulations that involve distance measurements, where the Migliore neuron model is used instead (Migliore and Migliore, 2012). Since the Izhikevich model has no geometry component, measurements with respect to distance cannot be performed using the Izhikevich model. The Migliore model, on the other hand, is a reconstruction of a hippocampal CA1 neuron, which consequently has dendritic geometry that can be used for our simulation study. In the neuron model, we simulate synaptic activity which results in firing of the cell; the action potential is then backpropagated to the spine model, which we use as input to either the mechanistic or the IO model. The second input, i.e., NMDA-R channel conductance, is a critical measure of pre- and postsynaptic activity; we determine the total NMDAr conductance based on both pre- and post-synaptic activity of the spine.

In our platform, opening of the NMDA receptor channel model: (1) Allows calcium influx into the spine, and (2) produces postsynaptic currents, which are passed to the neuron model and influences the postsynaptic potential. We chose the NMDAr channel conductance as an input parameter to the input-output model to account for the calcium influx while still allowing NMDAr channels to influence postsynaptic activity. The output response of the IO model is calcium concentration, calibrated using the calcium concentration obtained with the mechanistic model. Thus, the influence on calcium dynamics of all other components besides the NMDAr model are captured in the IO model. The use of the IO model then allows us to model complex nonlinear calcium dynamics without requiring the large number of components that would otherwise be necessary when using a mechanistic model.

To describe the structure of the IO model, we first begin with a description of the SISO Volterra series:

(12) $$\begin{array}{l} u_{SISO}\left(t\right)=\;c_{0}+\overset{L}{\underset{j=1}{\sum }}c_{1}\left(j\right)v_{j}\left(t\right)+\overset{L}{\underset{j_{1}=1}{\sum }}\overset{j_{1}}{\underset{j_{2}}{\sum }}c_{2s}\left(j_{1},j_{2}\right)v_{j_{1}}\left(t\right)v_{j2}\left(t\right)\\ +\;\overset{L}{\underset{j_{1}=1}{\sum }}\overset{j_{1}}{\underset{j_{2}=1}{\sum }}\overset{j_{2}}{\underset{j_{3}=1}{\sum }}c_{3s}\left(j_{1},j_{2},\;j_{3}\right)v_{j_{1}}\left(t\right)v_{j2}\left(t\right)v_{j3}\left(t\right) \end{array}$$

(13) $$\begin{array}{l} v_{j}\left(t\right)=\;\overset{M}{\underset{\tau =0}{\sum }}b_{j}\left(\tau \right)x\left(t-\tau \right) \end{array}$$

Where *u*_*SISO*_ is the single input Volterra series up to 3rd order. L refers to the total Laguerre functions in the SISO model, and *c*_0_, *c*_1_, *c*_2*s*_, and *c*_3*s*_ refer to the coefficients associated with the 0th, 1st, 2nd, and 3rd order response of the series, respectively. *v*_*j*_(*t*) is the convolution of the input to the IO model with the basis function; *x*(*t*−τ) is the input to the IO model; and *b*_*j*_(τ) is the j-th basis function. *M* is the memory window of the IO model, set to 5 s. The coefficients are determined during the training process to best fit the nonlinear response of calcium dynamics. For our case, we use the Laguerre basis functions for our model. The Laguerre equations are used for their orthogonality and convergence properties, which are characteristic of many biophysiological systems (Berger and Song, 2010; Ghaderi et al., 2011).

The structure of the Volterra functional series in the MISO model is similar to the SISO model. In the case of two inputs, the series consists of the summation of two SISO model components that account for two different inputs, then adding a cross-kernel component accounting for possible nonlinear interactions that may occur due to the presence of two inputs. The equations become thus:

(14) $$\begin{array}{l} u_{MISO}\left(t\right)=\;c_{0}+u_{1}\left(t\right)+u_{2}\left(t\right)\\ +\overset{L_{1}}{\underset{k_{1}=1}{\sum }}\overset{L_{2}}{\underset{k_{2}=1}{\sum }}c_{2r}\left(k_{1},k_{2}\right)v_{k1}^{u_{1}}\left(t\right)v_{k2}^{u_{2}}\left(t\right)\\ +\overset{L_{1}}{\underset{k_{1}=1}{\sum }}\overset{L_{2}}{\underset{k_{2}=1}{\sum }}\overset{L_{1}}{\underset{k_{3}=1}{\sum }}c_{3r_{1}}\left(k_{1},k_{2},k_{3}\right)v_{k1}^{u_{1}}\left(t\right)v_{k2}^{u_{2}}\left(t\right)v_{k3}^{u_{1}}\left(t\right)\\ +\overset{L_{1}}{\underset{k_{1}=1}{\sum }}\overset{L_{2}}{\underset{k_{2}=1}{\sum }}\overset{L_{2}}{\underset{k_{4}=1}{\sum }}c_{3r_{2}}\left(k_{1},k_{2},k_{4}\right)v_{k1}^{u_{1}}\left(t\right)v_{k2}^{u_{2}}\left(t\right)v_{k4}^{u_{2}}\left(t\right) \end{array}$$

(15) $$\begin{array}{l} v_{j}^{u_{i}}\left(t\right)=\;\overset{M}{\underset{\tau =\;0}{\sum }}b_{j}\left(\tau \right)x^{u_{i}}\left(t-\tau \right) \end{array}$$

Here, *u*_*MISO*_ is the equation for the multi-input Volterra series, and *u*_1_ and *u*_2_ are the single input Volterra series based on *u*_*SISO*_ with the inputs the postsynaptic potential (*V*), and the NMDA receptor conductance (*g*_*totalNMDA*_), respectively. The cross kernel components involve the number of Laguerre functions *L*_1_ and *L*_2_ from the first and second SISO components, respectively, and at all orders. $v_{k}^{u}\left(t\right)$ is the associated basis functions convolved with inputs: postsynaptic potential (*V*), and the NMDA receptor conductance (*g*_*totalNMDA*_). *c*_*r*_ is then the associated coefficients for these terms. As a result, the nonlinearities influenced by having two different inputs are considered.

The IO model must be trained to tune its parameters and minimize the error with respect to the original system—in this case, the mechanistic model. The training process consisted in using the response of the original mechanistic model to random Poisson train events given at both low average frequency (2 Hz) and high average frequency (10 Hz), with a total of 1,000 events to ensure a wide range of activity. The low average frequency of 2 Hz was chosen to reflect the typical firing rate of hippocampal CA1 and CA3 neurons during resting state (Berger et al., 1988). Meanwhile, the high average frequency of 10 Hz is used to account for situations with higher levels of neuron activity in physiological conditions (Ranck, 1973). The coefficients associated with the basis functions were determined through the pseudoinverse matrix multiplication, and the optimal decay values associated with the Volterra series were estimated using gradient descent to find the parameters that resulted in the lowest root mean square error value in the comparison between the estimated and the actual result from the original model.

#### Platforms and computational tools

The mechanistic calcium model is described in the Systems Biology Markup Language (SBML) and was constructed using CellDesigner, a visual diagram editor for SBML (Funahashi et al., 2008). Our models were then run in MEMORY, a python-based platform designed for synapse-based simulations using the libSBML library and the simulation engine libroadrunner (Somogyi et al., 2015). In our studies which involve measuring calcium and dendritic potential based on distance from the soma, we required a neuron model with a detailed dendritic structure where synapses can be placed along the dendritic arbor. To achieve this, we use the CA1 pyramidal cell model designed by Migliore (Migliore and Migliore, 2012) simulated within NEURON cell simulation platform (Carnevale and Hines, 2006). The IO calcium model first required the coefficients to be determined, which were calculated within MATLAB; afterwards, the model was implemented into the MEMORY platform to run for simulation. Unless otherwise indicated, the input stimulus provided to synapses during simulation were Poisson 2 Hz randomized input trains, where each synapse was provided a unique randomized input. All simulations were conducted on a computer using the Fedora OS, with Intel quad-core 2.67 GHz processor and 8 Gb RAM.

## Results

### Calcium dynamics calibration and validation with published experimental data

**Figure 4:**
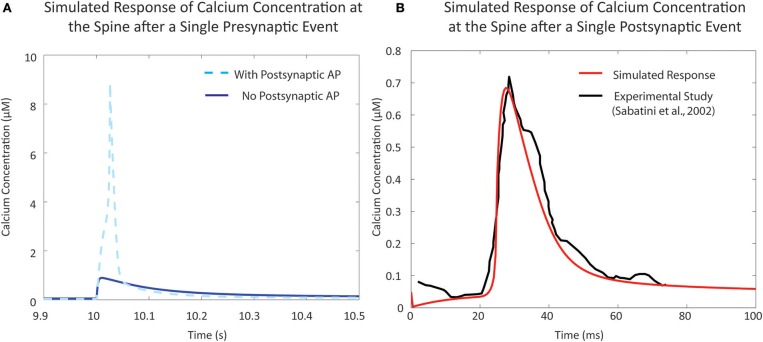
Validation of single event responses of the mechanistic model of calcium concentration at the postsynaptic spine. **(A)** The response of the postsynaptic spine model to a single presynaptic event with an elicited action potential (in light blue, dashed) that reaches ~9 μM, and without any resulting action potential (in blue, solid) that reaches ~1 μM, in line with expected amplitudes that have been reported in experimental studies and reviews (Sabatini et al., 2002; Higley and Sabatini, 2012). Neurotransmitter release following a presynaptic event opens NMDA receptor channels, causing calcium influx; the degree of influx is also dependent on membrane potential due to the voltage-dependent magnesium block. **(B)** Response to a single postsynaptic backpropagation event by the postsynaptic spine model (in red) in comparison to reported calcium response seen in the study by Sabatini et al. (2002) (in black). Voltage-dependent calcium channels (VDCC) open in response to a change in calcium levels—the result shown here is the response due to low-voltage activated VDCC-T type channels in the postsynaptic spine model.

In the resting phase, the average cytosolic calcium concentration in spines is typically kept at around ~50 nM, maintained by various pumps and buffers (Higley and Sabatini, 2012). Calcium influx occurs during activation of the various channels present on the spine. There are two major sources of calcium influx: Glutamate-dependent calcium influx, where calcium flows into the spine via NMDA receptor channels, and Voltage-dependent calcium influx, primarily through voltage-dependent calcium channels. For calibration of the mechanistic calcium model at the spine, we consider two scenarios: (1) calcium influx due to presynaptic activation, where the presynaptic terminal releases glutamate in response to a presynaptic action potential, and (2) calcium levels when the postsynaptic neuron is fired, leading to a backpropagating action potential (bAP). The response in (1) has been measured both in the presence and absence of postsynaptic depolarization (Sabatini et al., 2002; Higley and Sabatini, 2012). Our calcium model has a response of approximately 9 and 0.9 μM in the presence and absence of postsynaptic depolarization, respectively, which is in line with the experimental data presented in the literature (Figure 4A). Similarly, calcium levels in response to bAP were simulated and compared to calcium levels measured in Sabatini et al. (2002) (Figure 4B). The backpropagation factor assumed that the spine was a distance of ~150 μm from the soma, similar to the synapses recorded in the literature. The simulated results showed an amplitude of approximately 700 nM, comparable to measurements from Sabatini et al. (2002).

### Calcium fluctuations vary according to the inter-spike intervals between presynaptic and postsynaptic activity of the neuron

**Figure 5:**
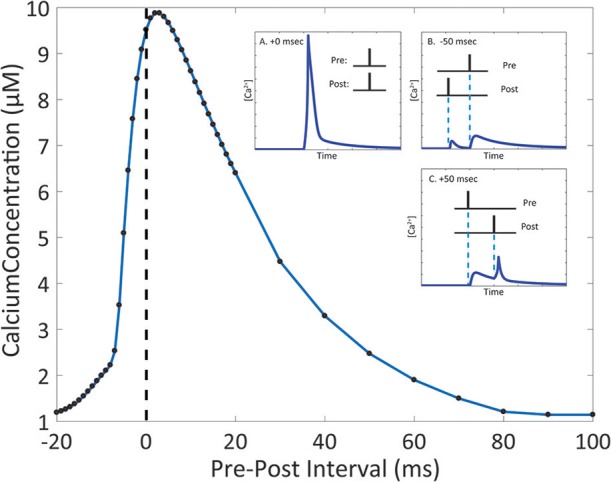
The magnitude of the calcium response changes depending on the interval between presynaptic and postsynaptic events. Timing interval between presynaptic and postsynaptic events can influence the amplitude of calcium dynamics in the mechanistic model of calcium at the postsynaptic spine. Pre-Post interval is defined as the amount of time in milliseconds the postsynaptic event (presynaptic neuron activation, NT release is triggered) occurs after the presynaptic event (postsynaptic neuron activation, the action potential is backpropagated to the spine). Black dots indicate measured maximum calcium response for simulations with the designated pre-post interval. In the inset, A., B., and C. show the simulated calcium profile in response to different pre-post intervals. The change in maximum calcium amplitude reflects the kinetics of the NMDAr channel dynamics, where the voltage dependent magnesium block results in the nonlinear behavior of the calcium response.

Due to the number of mechanisms in place, the integration of glutamate-dependent calcium influx and voltage-dependent calcium influx leads to further complex dynamics in calcium concentration at the spine. When the membrane potential increases as a result of postsynaptic events, the magnesium block is removed in the NMDA receptor channel pore, increasing the influx of ions when the receptor is activated (Jahr and Stevens, 1990; Ambert et al., 2010). In our model, we measured the maximum amplitude of calcium concentration at varying pre-post intervals to determine its influence on calcium dynamics in the spine (Figure 5). Through the study, we found that the highest calcium concentration peak reached was 9.88 μM, when the postsynaptic event occurred 3 ms after the presynaptic event. It was also noted that for pre-post intervals, where the presynaptic event precedes the postsynaptic event, there is a notable increase in the magnitude in comparison to the calcium response when there is only presynaptic activation with no postsynaptic activation. The increase in maximum calcium amplitude is present when the pre-post interval ranges from 0 to 80 ms; beyond 80 ms, there appears to be no significant change in maximum amplitude compared to presynaptic activation only. In the case of post-pre intervals, where the presynaptic event follows the postsynaptic event (corresponding to negative delays in Figure 5), calcium amplitude begins rising sharply starting at 20 ms all the way to 0 ms. It is interesting to note that while our model does not account for synaptic plasticity, the pre-post interval time-scale dependency of calcium amplitude on pre-post intervals resembles the well-known spike timing dependent plasticity (STDP) curve presented by Bi and Poo (2001), particularly in regards to synaptic strengthening when post-synaptic activation follows pre-synaptic activation. The correlation in timescale dependence between the STDP curve and the presented results from the mechanistic calcium model suggests the mechanistic model can be considered a plausible model basis for a plasticity model in the future.

### Postsynaptic calcium activity depends on the distance between the synapse and the soma

**Figure 6:**
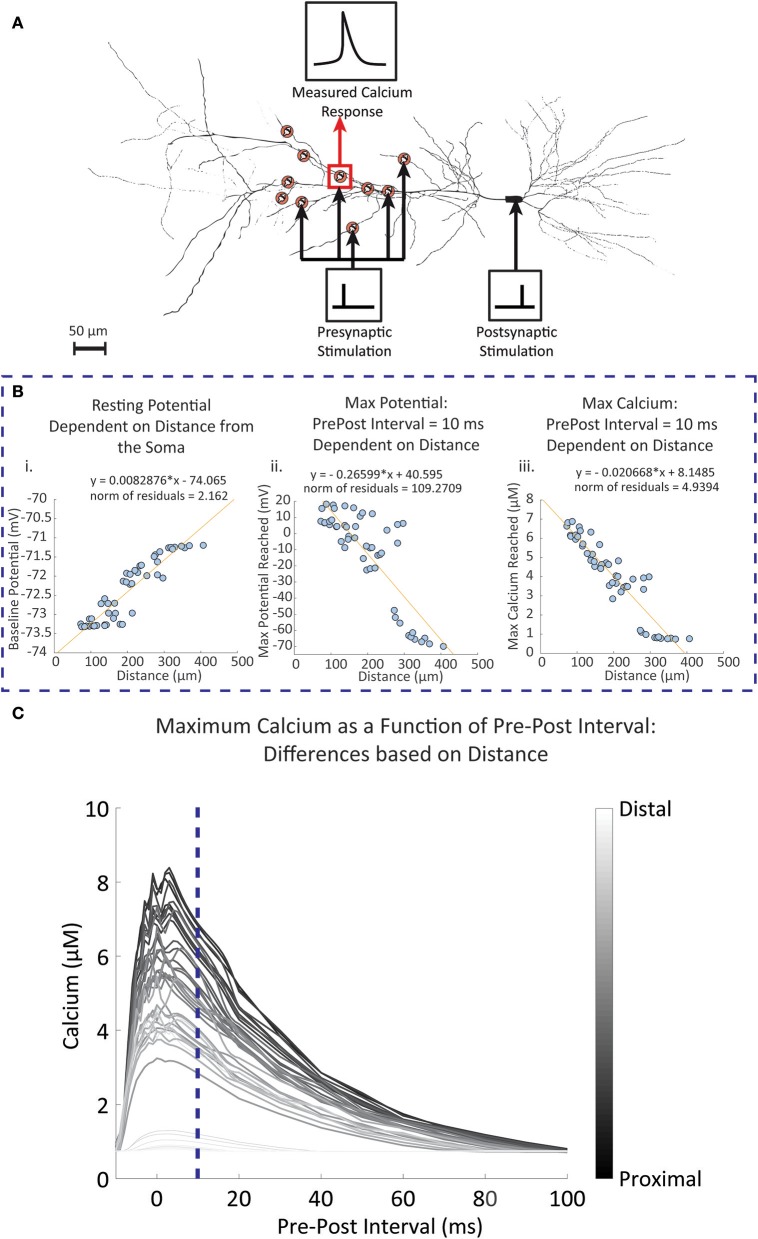
Simulated results of dendritic potential and maximum calcium amplitudes as a function of distance from the soma. Fifty random locations were chosen within the stratum radiatum sections of a pyramidal CA1 cell model by Migliore and Migliore (2012). **(A)** Diagram of the simulation protocol for stimulating and measuring calcium levels for synapses located at different distances from the dendrite. Presynaptic stimulation indicates simulated presynaptic release on the respective synapse, while postsynaptic stimulation indicates simulation of the soma, inducing a backpropagating action potential. **(B)** Synapses were stimulated with a presynaptic release event at time 0, followed by a simulated current injection into the soma of the neuron 10 ms following the presynaptic event, triggering an action potential and invoking backpropagation. (i) The dendritic resting potential values from the 50 locations with respect to distance. On average, the potential is slightly higher in distal than in proximal locations by approximately 2 mV. (ii) In contrast to the baseline potential, the maximum potential reached with the backpropagation of a single postsynaptic spike becomes more attenuated when further away from the soma. (iii) The influence of the attenuation is seen in the calcium measurements, where maximum calcium is reduced when moving further away from the soma, with 300 μm and beyond the backpropagation becomes negligible. **(C)** The pre-post interval was varied between simulations, and the maximum calcium response was measured for each simulation at each synapse location. The darker lines indicate calcium responses from synapse locations closer to the soma, while lighter lines are responses from locations further away from the soma. The blue dotted line is where the pre-post interval is 10 milliseconds, reflecting results that are seen in (**B**i) through (**B**iii).

The experimental studies conducted by Sabatini et al. (2002) observed synapses located approximately 150 μm from the soma. Studies have shown that backpropagation signal properties of the synapse and dendrite can change depending on distance (Golding et al., 2001). To observe the influence of distance on the calcium response, we used the CA1 pyramidal neuron model by Migliore and Migliore (2012) to simulate 50 randomly placed synapses on the stratum radiatum, with the closest synapse having a distance of 72.62 μm and the furthest synapse has a distance of 407.03 μm; these values are close to the minimum and maximum range specified for the stratum radiatum (Megías et al., 2001). When synapses were randomly placed on the pyramidal cell model, we measured the dendritic diameter of the synapse locations. It was found that 45 (90%) of the synapse locations had a diameter of 0.5 μm. This corresponds to the measured diameters of the thin dendrites located in the stratum radiatum in experimental findings (Megías et al., 2001). Other diameters were 2, 2, 1.2, 1.2, and 0.18 μm. Our simulations from these synapses showed no influence of the diameter on our measured results. In our first set of simulations, the synapses were stimulated with a single presynaptic event, then a fixed single postsynaptic event following 10 ms afterwards. Figure 6A shows a schematic of the simulation setup. In Figure 6B, we summarize the results where for each synapse we consider (Figure 6Bi) the resting potential, Figure 6Bii maximum amplitude of the backpropagating action potential during stimulation with a pre-post interval of 10 ms, and Figure 6Biii maximum calcium response during stimulation with a pre-post interval of 10 ms. We note that both the resting potential from Figure 6Bi and the max bAP from Figure 6Bii are properties inherent to the neuron model described by Migliore and Migliore (2012). For Figure 6Biii, we simulate calcium dynamics at the spine using our mechanistic calcium model described in this manuscript. In Figure 6C, we repeated the simulations with different pre-post intervals and measured the maximum calcium response at the spine of each synapse location.

Our simulation results indicated that baseline potential is slightly increased in distal dendrites than in proximal dendrites, with a range lying between −73.4 mV and −71 mV. Such a difference appears rather small, but still constitutes a notable trend with respect to distance. Differences up to 2 mV between somatic and dendritic resting potential have been observed experimentally, and our model falls in line within these constraints (Golding et al., 2001). Conversely, the maximum potential reached after pre-post event stimulations decreases with respect to distance; proximal dendrites are more likely to reach a higher maximum potential than distal synapses.

Observations of the calcium concentration levels at the postsynaptic spine indicated that at a pre-post interval of 10 ms, the amplitude of the calcium concentration peak in spines decreased as distance with the soma increased. Again, beyond 300 μm we found that calcium amplitudes do not extend much beyond 1 μM, likely due to the reduced influence of the postsynaptic activity. Subsequent simulations where the pre-post intervals are changed demonstrated that the influence of the timing between presynaptic and postsynaptic events is more prominent in proximal synapses than in distal synapses. In particular, there no longer appears to be any dependency of the max calcium response on the pre-post interval timing for the synapses farthest away from the soma. These simulations are in line with studies on the influence of distance on STDP, where it was found that backpropagation induces LTP more commonly in proximal synapses, while at distal synapses LTD occurs more frequently in response to the same backpropagating potential (Sjöström and Häusser, 2006).

Results presented here suggest that the bAP is significantly attenuated in distal spines to the degree that a single pre-post events does not trigger much calcium influx. At the time of writing, experimental data of spine calcium levels based on distance have not been documented in the literature. However, the results presented on backpropagation are in line with what has been observed in experimental studies, such as Golding et al. (2001), where it was observed the bAP amplitude is reduced when further away from the soma, and especially beyond 300 μm. The reason for attenuation is likely due to two factors: (1) changes in active conductance with respect to distance from the soma, where at distal dendrites there is a higher density of potassium channels and low density of calcium and sodium channels (Bikbaev et al., 2016); and (2) the branching of the dendritic arbors, which has also been seen to contribute to the attenuation of the bAP (Golding et al., 2001). In distal synapses, there may be other mechanisms at play that may more prominently influence signaling and plasticity to compensate for attenuated bAP, such as modulation by glial cells and neurotransmitters (acetylcholine, brain derived neurotrophic factor, dopamine noradrenaline) (Edelmann et al., 2017).

### A third order, multi-input input-output calcium model closely replicates the response of the mechanistic calcium model at lower frequency

Each individual component in the mechanistic calcium spine model has its own degree of computational complexity, and the integration of all the components also compounds the overall computational burden. As a result, simulation of the calcium dynamics for a larger number of spines becomes increasingly difficult. We demonstrate here the use of an input-output model based on the Volterra functional series that reduces computational cost of simulating calcium dynamics. The output of the model is calcium concentration. The inputs to the proposed model are membrane potential and NMDA receptor conductance. The NMDA receptor model is the only component in the calcium dynamics model that we consider outside of the IO calcium model, since an IO NMDA model had been developed before and can be utilized in its place (Hu et al., 2015). In brief, the IO NMDA model is a single-input-single output model which uses the Volterra series with Laguerre basis functions to predict the open state probability of the NMDA receptor channel (“Open” from Supplementary Figure 1). The open state probability is then used to calculate the conductance based on the magnesium blockade equation (Equations 3–5). However, for the purposes of consistency, in our simulations we use the kinetic NMDA receptor model to properly compare results only between the IO calcium model and mechanistic calcium model. Training the IO model requires keeping track and replicating calcium concentration profiles from the mechanistic model in three separate conditions. First, when only presynaptic stimulation is applied; then, when only postsynaptic stimulation is applied (back-propagated action potential); and finally, when both presynaptic and postsynaptic stimulations are applied to the spine. For each type of stimulation, we used Poisson random interval trains for 1,000 events at 2 Hz and 1,000 events at 10 Hz for a total of 2,000 events, as each input. This gives us a total of 8,000 events for the model to be trained on: 4,000 events in total for presynaptic stimulation, and 4,000 events total for postsynaptic stimulation. The large number of events at lower and higher frequencies gives the IO model an adequate range of nonlinear dynamics to be trained on. At the end of the training phase, the root mean square (RMS) difference is calculated and normalized to the maximum value and minimum of mechanistic model response. The difference between the mechanistic model and the trained IO model was 6.79% with the given training data.

**Figure 7:**
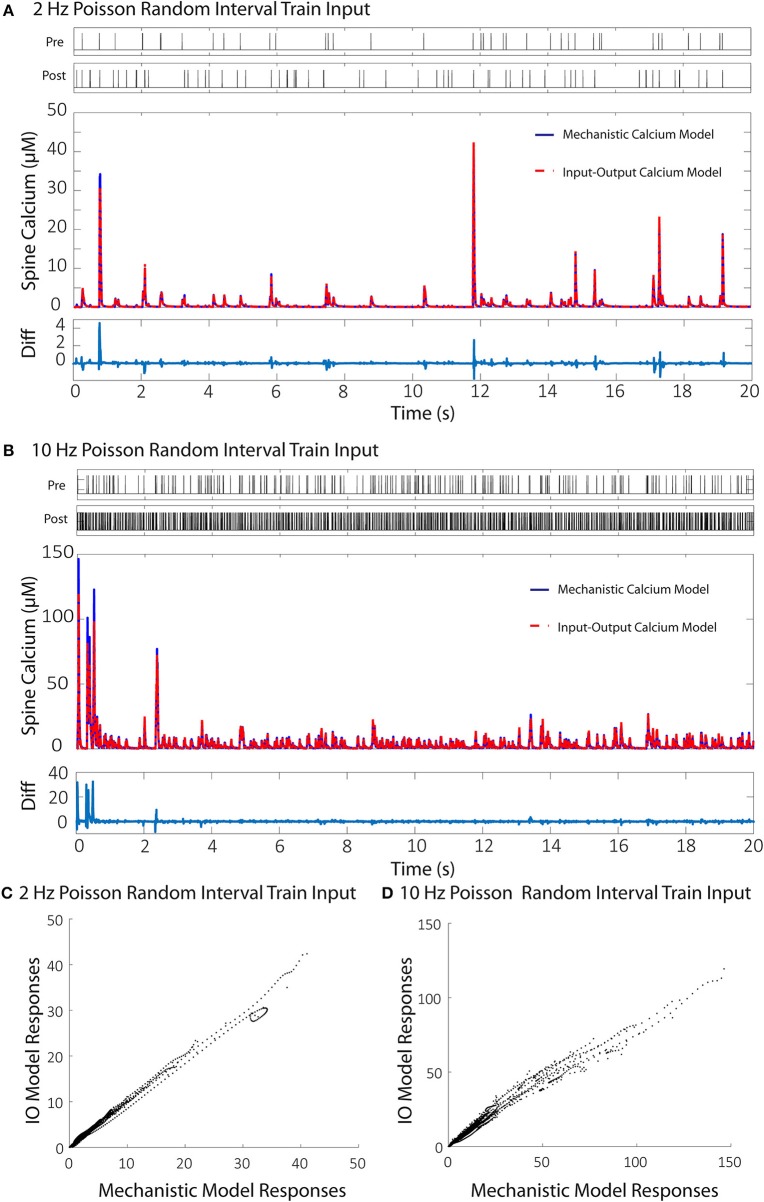
Comparisons between the calcium responses from the mechanistic model and the IO calcium model. **(A,B)** Shows the responses of the mechanistic model (in blue) and the trained IO calcium model (in red) over the course of 20 s given Poisson randomized presynaptic and postsynaptic events with an average of 2 and 10 Hz, respectively. The difference between the mechanistic and the IO model are plotted beneath each response. The calculated RMS difference between the two models is 8.15% for the 2 Hz response and 16.9% for the 10 Hz response. **(C,D)** Shows a direct comparison between the calcium response values from the mechanistic model (x axis) and the IO calcium model (y axis) from the 2 and 10 Hz responses, respectively.

**Figure 8:**
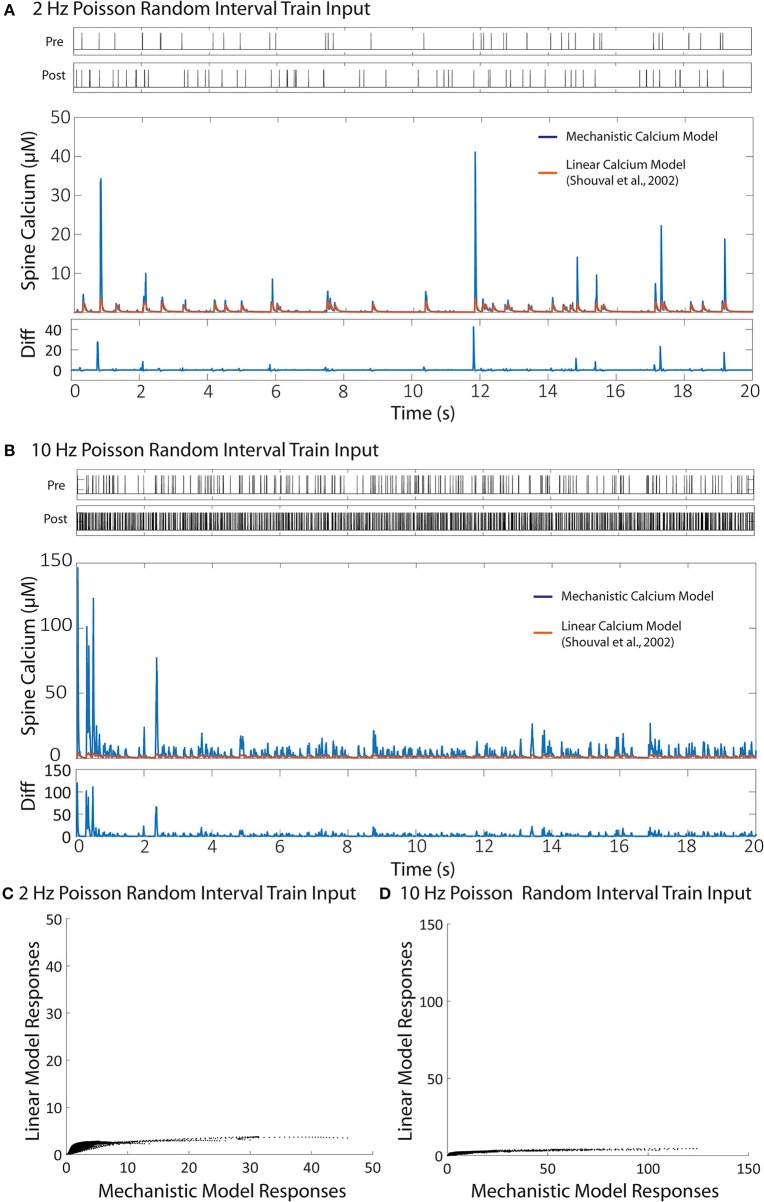
Comparison between the linear calcium model (Shouval et al., 2002) and the mechanistic calcium model. **(A,B)** Shows the responses of the mechanistic model (in blue) and the linear calcium model (in orange) over the course of 20 s given Poisson randomized presynaptic and postsynaptic events with an average of 2 and 10 Hz, respectively. The root mean square difference between the two models is 80.52% for the 2 Hz average response, and 89.75% for the 10 Hz average response. **(C,D)** Shows a direct comparison between the calcium response values from the mechanistic model (x axis) and the linear calcium model (y axis) from the 2 and 10 Hz responses, respectably.

We then validated the trained IO model with a naive train of presynaptic and postsynaptic stimulations for both 2 and 10 Hz and compared the results with the ones obtained with mechanistic model. The trained IO model was found to be more accurate at lower frequencies: the validation error at 2 Hz was 8.15%, while the error for 10 Hz reached 16.9% (Figure 7). We have also provided a comparison between the mechanistic model response and the response from the linear calcium model by Shouval et al. (2002), which is presented in Figure 8. The linear calcium model is presented as:

(16) $$\begin{array}{l} \frac{d\left[Ca^{2+}\right]}{dt}=I_{NMDA}\left(t\right)-\left(\frac{1}{\tau_{Ca}}\;\left[Ca^{2+}\right]\right) \end{array}$$

[*Ca*^2+^] represents calcium concentration. *I*_*NMDA*_(*t*) is the contribution of calcium current provided by the NMDA receptors; in our simulations, we used the NMDAr kinetic rate model described by Erreger et al. (2005) to determine calcium current from NMDA receptors. This is the same NMDAr model used for our mechanistic calcium model and IO calcium model. τ_*Ca*_ is the time constant for calcium decay in the linear model. As a result, all nonlinearities associated with NMDAr kinetics are also being accounted for in the simulation with the linear calcium model. We calibrated the parameter to 20 ms, which best approximates the decay of the first order response to the mechanistic model. After calibration, we simulated Poisson random input events with an average frequency of 2 and 10 Hz to the linear model to compare with the mechanistic calcium model response. The difference is shown in Figure 8. The root mean square difference between the two models is very large: 80.52% for the 2 Hz average response, and 89.75% for the 10 Hz average response. This demonstrates that, even with the nonlinear dynamics of the NMDAr model accounted for, considerable nonlinearities in the mechanistic calcium model exist that cannot be replicated in a linear calcium model. In particular, the results from the linear model significantly undershoots the calcium dynamics seen in the mechanistic model as demonstrated in Figures 8C,D. Because the nonlinear dynamics of the NMDA receptor channel have been accounted for, these differences in nonlinearity are more likely a result of the buffers, VDCC, and calcium influx dynamics simulated within the mechanistic model but not in the linear model. Meanwhile, the nonlinear dynamics are reproduced in the IO model, where the RMS error was shown to be much lower.

**Figure 9:**
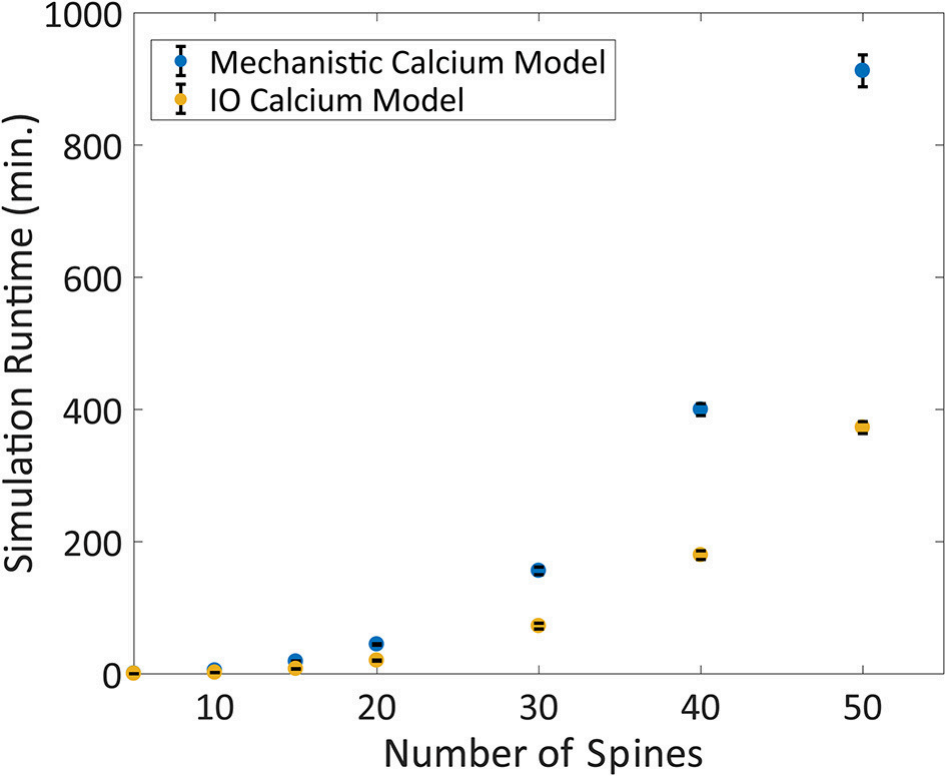
Comparison between the Mechanistic and IO model runtime vs. number of spines. Each spine represents the kinetics for synaptic transmission as well as calcium dynamics, which is either represented by the mechanistic calcium model or the IO calcium model. Benchmarking was conducted at 5, 10, 15, 20, 30, 40, and 50 spines, and simulations were repeated 10 times to derive the standard deviation of the runtimes. For each simulation, a 2 Hz Poisson random interval train input was given to all synapses. Overall, the IO spine model required on average a little less than half the runtime needed to finish a simulation compared to the mechanistic model.

Following validation, the computational time to run the IO model and the mechanistic model was determined based on number of spines with 2 Hz Poisson random interval train inputs. We find that the IO model finished the simulations faster than the mechanistic model, where the runtime of the IO model required around half the time to finish a simulation compared to the mechanistic model (Figure 9).

**Figure 10:**
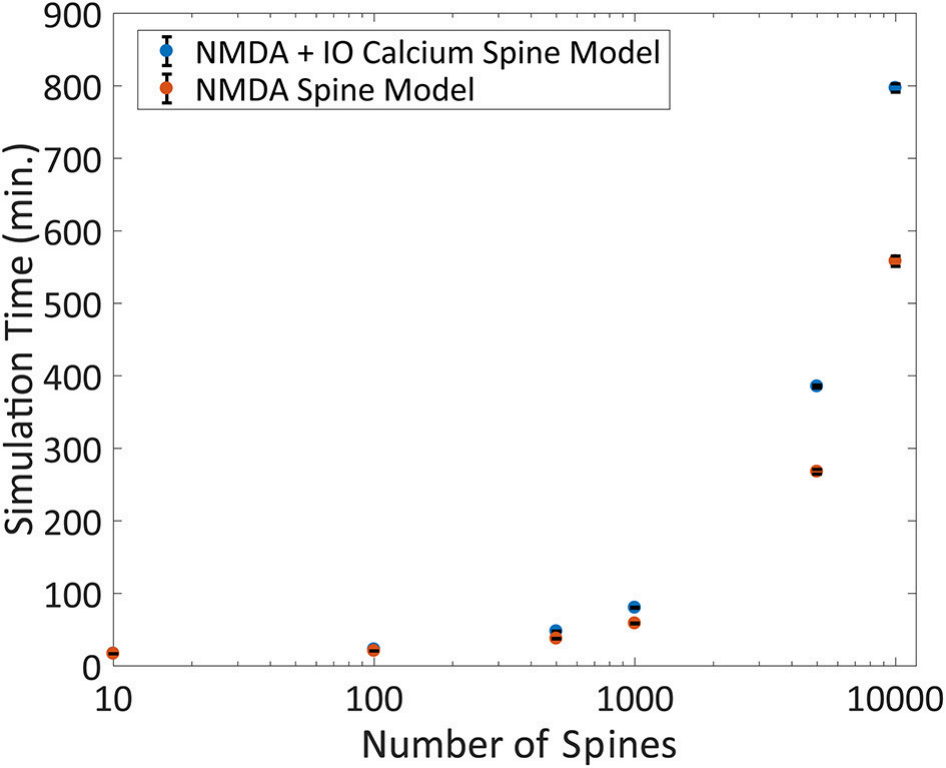
Benchmarking the IO model within the NEURON simulation platform. The IO model was re-implemented as a module file usable within the NEURON platform and simulated on a compartmental neuron model for 20 simulated seconds at 0.1 ms timesteps. The number of spines was varied for each simulation ranging from 10 to 10,000, where a spine was defined as a kinetic NMDA 8 state model, either with or without the IO calcium model and the simulation runtime is plotted here. Each simulation was repeated 10 times each to derive the standard deviation, shown as error bars in the figure.

Another advantage of the IO calcium model is that the framework of the input-output model is easily implemented and adaptable to other simulation platforms. To test the performance of the IO calcium model simulated as an embedded mechanism within the NEURON engine, we adapted the IO model into a module file for the NEURON platform and compared cell level simulations based on number of spine instances. Two types of models were simulated with different spine configurations: (1) spines using both an NMDA 8 state model and the IO calcium model, and (2) spines with only the NMDA 8 state model and no IO calcium model. Simulation protocols with 10, 100, 500, 1,000, 5,000, and 10,000 spine instances were conducted; simulations were run in fixed time step of 0.1 ms, a randomized poisson input train of 2 Hz frequency, and the overall simulated time period is for 20 s. Simulation times were then benchmarked to determine how much of a computational burden is added when including IO calcium model within spines. Simulations were repeated 10 times each to derive the standard deviation in the simulation time. Results are shown in Figure 10. Our simulations concluded that at 10,000 spines, the computational burden increases from 9.3 to 13.3 h. For details on the variation in the benchmarks, the standard deviation of the benchmarking data from Figures 9, 10 are plotted in Supplementary Figure 4. While the increased required simulation time is not insignificant, the IO framework still gives a viable option for simulating complex postsynaptic calcium dynamics on a larger scale—with numerous spines on a neuron or a neuron network.

## Discussion

This article describes the development and simulation of a model of the postsynaptic calcium concentration in the spine. The model presented is an integration of various mechanisms which shape the dynamics of calcium concentration at the postsynaptic spine, comprising elements that contribute to calcium influx, calcium extrusion, and buffering. Experimental studies on spine signaling have focused on calcium more than any other signaling molecule within active spines (Higley and Sabatini, 2012), as calcium dynamics and its effectors (NMDA, VDCC, etc.) have been repeatedly shown to strongly influence plasticity and learning. Calcium has also been implicated as a role player in neurodegenerative diseases such as Alzheimer's Disease (Khachaturian, 1994; Alberdi et al., 2010). Our goal in modeling calcium is to: (1) explore mechanisms and details underlying calcium dynamics that would otherwise be difficult to achieve with experimental studies alone (i.e., influence of pre-post timing and distance on spine calcium, where the researcher must take multiple time measurements at multiple spine locations, would be difficult to measure in experimental setups), and (2) reduce computational complexity of the calcium model to enable multi-scale simulations. We have presented a viable model which is supported by experimental data. We configured the model to replicate a particular type of synapse—a glutamatergic CA3-CA1 synapse of a pyramidal cell neuron; it incorporates the nonlinear dynamics that result from interactions between the components that contribute to spine calcium concentration.

Beyond experimental validation, we presented simulated results with the mechanistic model which show changes in spine calcium activity as a function of presynaptic and postsynaptic intervals—a standard protocol for inducing spike-timing dependent plasticity. In STDP, the weight of a synapse changes after repeated identical pre-post stimulations at given pre-post intervals. In hippocampal CA1 glutamatergic spines, intervals where presynaptic stimulation precedes postsynaptic stimulation induce synaptic potentiation, with the strength of the potentiation inversely proportional to the interval distance between the pre- and post-stimulation (Bi and Poo, 2001). Our model demonstrates that similarly, calcium influx is significantly amplified when presynaptic stimulation precedes postsynaptic stimulation, and that the amplitude is also inversely proportional to the interval size. Many plasticity associated signaling cascades are activated by calcium—for example, AMPA receptor upregulation into the spine is a known indicator for synaptic strengthening (Zhabotinsky et al., 2006). This process is initiated by spine calcium binding with CaMKII and triggering secondary messenger pathways. Likewise, recruitment of actin will lead larger spines—this, too has been associated with calcium interaction (Araya, 2014).

It is thought that the major influence on the calcium brought about by presynaptic/postsynaptic interactions is the NMDA receptor channel kinetics, but we have demonstrated in our simulations that the NMDAr channel alone is not the sole contributor of the nonlinear dynamics of calcium in the spine. The role of NMDAr in synaptic activity is considerably important: it has been shown experimentally that NMDA contributes to synaptic plasticity and LTP (Sakimura et al., 1995; Grover et al., 2009; Larson and Munkácsy, 2015). Many LTP models are based around this hypothesis, where NMDAr models are used to represent calcium influx, and repeated stimulation leads to calcium induced plasticity (Shouval et al., 2002; Standage et al., 2014). However, the NMDA representation in such models is a simple, linear representation where the NMDA-based calcium influx is represented as a ratio proportional to the bAP. This poorly reflects on nonlinear calcium dynamics in two ways: (1) the simplified version of NMDAr dynamics ignores important dynamical features that are known to be associated in NMDAr channels, such as desensitization (Mayer et al., 1989), which is included as a state in the NMDAr kinetic model used in our mechanistic calcium platform, and the magnesium blockade (Calabresi et al., 1992) which is instead roughly approximated using a BPAP curve; (2) there is no influence or contribution from other elements or properties from the spine, which can drastically alter the calcium response. Meanwhile, our model integrates validated channel kinetics within the confines of the spine compartment. Thus, our mechanistic model can consider the nonlinear aspects of calcium influx which are influenced by NMDAr channels, along with other channels, pumps, and buffers that regulate spine calcium concentration—all of which influence observed calcium levels at the spine. Especially to note is the spine volume and the buffers within the spine. Changes to volume can result in undercompensation (in larger volumes) or overcompensation (in smaller volumes) of calcium concentration, unless the mechanisms which govern calcium dynamics are scaled appropriately (O'Donnell et al., 2011). Furthermore, experimental evidence has shown that changes in volume also can result in change in AMPAr expression and upregulation (Noguchi et al., 2011), which is also important for synaptic plasticity. Such mechanisms revolved around volume are ignored in linear calcium concentration models, but can be studied in future simulations using the mechanistic model. Buffering provides another layer of complexity that will influence the amplitude and decay of the calcium response, yet there is no consideration for buffers in the linear calcium models. Our results demonstrate that, in the study of spine calcium, these components are important contributors to the nonlinear response of calcium.

The investigation into calcium dynamics at the postsynaptic spine is our latest study in the use of computational synaptic modeling platform to better understand synaptic activity and signaling: (1) We modeled the impact of astrocytic glutamate uptake (Allam et al., 2012) and ionotropic receptor distribution (Allam et al., 2015) on synaptic transmission in glutamatergic CA1 synapses; (2) Our synapse platform has been adapted to cellular and network levels in simulation (Bouteiller et al., 2011) to observe effects of nonlinear activity of synapses in a network simulation; (3) Large scale simulation models containing millions of neurons have also been developed within our research lab (Hendrickson et al., 2015); (4) and efforts had been made to adapt the complex nonlinear postsynaptic response of mechanistic synapses to large scale simulations using input-output modeling (Hu et al., 2015). Our modeling platform is consistently under expansion, with current projects considering the effects of modulators such as acetylcholine and how intracellular calcium stores influence metabolism and pump activity. The mechanistic and IO calcium model we describe in this manuscript expands our modeling platform to simulate not only synaptic transmission, but complex calcium dynamics as well. From here on, we plan to investigate and implement mechanisms that are based on spine calcium (the CaMKII signaling pathway, for example) and move to the next hierarchical level of calcium dynamics, the calcium response at the dendrite.

Expansion into large-scale, multi-scale modeling with complex biologically accurate synapse dynamics requires reduction of the computational burden while minimizing loss in accuracy. Spine calcium plays a key role in synaptic plasticity and influences communication between neurons, and understanding how calcium dynamics change network properties on a large scale can give us a better sense of the mechanisms that give rise to plasticity. Key downstream processes are influenced by the slightest changes in calcium dynamics (timing, magnitude, frequency, decay) (Evans and Blackwell, 2015). For example, an increase in spine calcium levels can activate signaling cascades that lead to either LTP or LTD induction (Lisman, 1989; Artola et al., 1990; Malenka and Bear, 2004); however, it is also observed in experiments that there is not a simple threshold that distinguishes when LTP or LTD occurs during calcium influx (Neveu and Zucker, 1996)—emphasizing even more the need of an integrated model of varying calcium dynamics, not just a linear model based on thresholds. Hence, we believe accurate representations of nonlinear calcium are required not only at the subcellular scale models, but on larger network-level models as well. Our model can provide an accurate reflection on the magnitude, duration, and location of spine calcium response—nonlinear dynamics that have been described to be more and more important for calcium based synaptic plasticity (Evans and Blackwell, 2015). Furthermore, neurodegenerative disease are often accompanied by an imbalance in calcium levels (Arundine and Tymianski, 2003). Nonlinear calcium models can be modified to represent pathological conditions, and multi-scale modeling can help identify the network level changes that occur with neurodegeneration and disease.

However, the computational cost of using many instances of the mechanistic calcium model in full exceeds the computational capacity of even the most recent high-performance computers; a method to improve computational efficiency is needed. Our previous work considers the use of the Volterra functional series to develop an IO model for the postsynaptic response to a synapse (Hu et al., 2015). Using the same input-output framework, we adapted this method to reduce the computational burden of modeling calcium dynamics. We have shown that using the IO model reduces the required simulation time by two to three-fold compared to the mechanistic model within our MEMORY platform. We also demonstrate that the IO framework can be easily adapted into other platforms such as NEURON, where, in our setup, 10,000 instances of the IO calcium model with the NMDAr kinetic model can be simulated on a complex, morphological, compartmental cell model, resulting in 1 h additional simulation time compared to the same protocol using the NMDAr kinetic model but without the IO calcium model. However, the current IO model as described has a limitation that must be addressed: it is limited to a 3rd order model, with higher order models requiring exponentially increasing memory requisites. Spine calcium dynamics become progressively more nonlinear when given higher frequency input, such as high frequency stimulation protocols often used in LTP induction. As such, other IO model frameworks are being investigated, such as the Laguerre-Volterra network structure (Geng and Marmarelis, 2016), as possible enhancements leading to even more efficient computational modeling of complex dynamic systems.

The concept of using computer simulations to study postsynaptic calcium dynamics is not new. There have been several computational models of spine calcium that have been developed previously, and their work has provided useful insights on the dynamics and importance of calcium (Shouval et al., 2002; Standage et al., 2014; Bartol et al., 2015). Generally, current calcium models are divided into two categories: (1) Phenomenological models which describe very few mechanistic aspects of calcium dynamics, but help understand its influence on synaptic plasticity and LTP (Shouval et al., 2002; Naoki et al., 2005; Zhabotinsky et al., 2006; Standage et al., 2014); and (2) detailed, stochastic models which describe calcium all the way down to each individual ion (Bartol et al., 2015). In (1), the models do not extensively consider calcium dynamics at length and may even consider calcium as a linear system. Instead, models from (1) evaluate the downstream effects of calcium on important synaptic processes such as plasticity. In contrast, the detailed calcium model in (2) considers a complete reconstruction of a small area on the CA1 pyramidal cell neuron, including specific channel densities and exact volume and shape reconstruction of a 6 × 6 × 5 μm^3^ cube of neuropil in a Monte Carlo based stochastic simulator. However, such a model is difficult to adapt outside of the scope of the reconstructed area and is computationally intensive, making it unsuitable for larger scale simulations. The models in this article help bridge this discrepancy: (1) the mechanistic model is capable of replicating complex non-linear interactions between the elements that shape spine calcium dynamics, and (2) the input-output model provides a method to simulate these complex calcium dynamics on a larger scale.

The spine is a constantly changing organelle as a result of development, plasticity, and/or pathological conditions. The current spine calcium model as described here represents only a snapshot of a particular spine, constrained by static parameters based on the averaged responses from experimental data. Future renditions of our model will not be limited to a single type of synapse. Our spine calcium model has potential to be adapted to varying physiological states (i.e., different morphologies and channel distributions) and pathological conditions (such as Alzheimer's disease). Furthermore, the model has potential applications in drug discovery, for *in silico* testing of compounds that modulate calcium (either directly or via channel/pump interactions). The calcium model is an expansion of the synapse model framework that is constantly being built upon to provide extensive and detailed multi-level models that can help explore the pathways and processes of that take place in the spine and influence synaptic plasticity, neuron communication, and pathological processes.

## Availability

Model scripts and code will be made available in the future on the EONS synaptic platform modeling site, www.synapticmodeling.com.

## Author contributions

EH and J-MB: concept and design of study; EH and AM: data acquisition; EH, DS, and J-MB: analysis and/or interpretation of data; EH and J-MB: drafting of the manuscript; CB, DS, J-MB, and TB: critical revision; EH, AM, CB, DS, J-MB, and TB: approval of the manuscript to be published.

### Conflict of interest statement

The authors declare that the research was conducted in the absence of any commercial or financial relationships that could be construed as a potential conflict of interest.
