# Supplementary material for: A Glutamatergic Spine Model to Enable Multi-Scale Modeling of Nonlinear Calcium Dynamics
Source: Front Comput Neurosci. 2018 Jul 27;12:58. doi: 10.3389/fncom.2018.00058 (PMC6072875; doi:10.3389/fncom.2018.00058)
Supplement: Supplementary file 1 [file Data_Sheet_1.DOCX]

Supplementary Material

**A glutamatergic spine model to enable multi-scale modeling of nonlinear calcium dynamics.**

**Eric Hu^1^ *, Adam Mergenthal^1^, Clayton S. Bingham^1^, Dong Song^1^, Jean-Marie Bouteiller^1^, Theodore W. Berger^1^,**

^1^Department of Biomedical Engineering, University of Southern California,

Los Angeles, CA, USA

*** Correspondence:** Corresponding Author: ehu@usc.edu

# Supplementary Figures and Tables

**
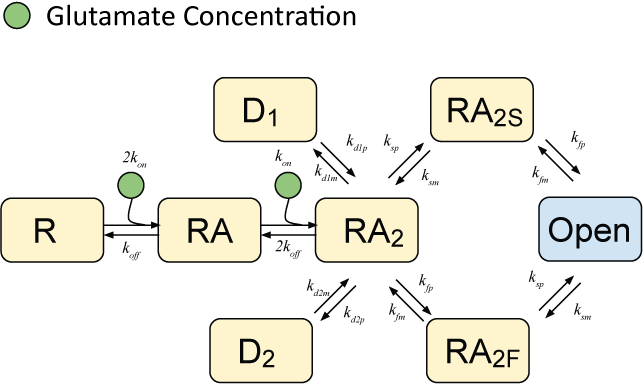
**

**Supplementary Figure 1.** The NMDA receptor model kinetic schema presented by (Erreger et al. 2005). Rate constant values are presented in Supplementary Table 1. In our model, each presynaptic event corresponds with neurotransmitter release, which reproduces a profile for glutamate diffusion that is described in (Allam et al., 2015). For every presynaptic event which occurs, the glutamate profile is modelled as:

$$Glu\left( r,t,Q,D,\delta\right)=\frac{Q}{4\pi\delta Dt}e^{\frac{r^{2}}{4Dt}}$$

*Glu* represents the glutamate inside the synaptic cleft. *r* indicates the radial distance of the receptors, set to 0.1 µm. *D* indicates the diffusion coefficient set to 0.4 µm^2^ / ms. Q is the number of neurotransmitter released in a single event, set at 3000. $\delta$ is the width of the cleft with the value 0.02 µm. All parameters relating to diffusion are directly derived from the study in (Allam et al., 2015).

| **Parameter** | **Value** |
| --- | --- |
| $k_{on}$ | 31.6 mM^-1^ms^-1^ |
| $k_{off}$ | 1.01 ms^-1^ |
| $k_{d1p}$ | 0.0851 ms^-1^ |
| $k_{d1m}$ | 0.0297 ms^-1^ |
| $k_{d2p}$ | 0.23 ms^-1^ |
| $k_{d2m}$ | 1.01e-3 ms^-1^ |
| $k_{sp}$ | 0.23 ms^-1^ |
| $k_{sm}$ | 0.178 ms^-1^ |
| $k_{fp}$ | 3.14 ms^-1^ |
| $k_{fm}$ | 0.174 ms^-1^ |

**Supplementary Table 1.** The NMDA receptor model kinetic rate constants from (Erreger et al. 2005).

| **Equations** | |
| --- | --- |
| $I_{Ca}=g_{VDCC}\cdot m^{2}\cdot h\cdot dvf$ | |
| $m=\frac{\alpha_{m}}{\alpha_{m}+ \beta_{m}}$ | |
| $h=\frac{\alpha_{h}}{\alpha_{h}+ \beta_{h}}$ | |
| $\frac{dm}{dt}=\alpha_{m}\left( 1-m \right)- \beta_{m}m$ | |
| $\frac{dh}{dt}=\alpha_{h}\left( 1-h \right)- \beta_{h}h$ | |
| $\alpha_{m}(V)=\frac{a(-V+b)}{\exp\left( \frac{-V+b}{10} \right)-1}$ | |
| $\beta_{m}\left( V \right)=c\cdot exp(-\frac{V}{d})$ | |
| $\alpha_{h}\left( V \right)=e\cdot exp(-\frac{V}{f})$ | |
| $\beta_{h}(V)=\frac{1}{\exp\left( \frac{-V+g}{10} \right)-1}$ | |
| $dvf=\frac{0.001}{0.001+\left[ Ca \right]_{i}}V\cdot(1-\frac{\left[ Ca \right]_{i}}{Ca_{o}}\exp\left( \frac{zV}{RT} \right))\cdot(\frac{1}{\exp\left( \frac{zV}{RT} \right)-1})$ | |
| **Parameter** | **Value** |
| a | 0.2 mV^-1^.ms^-1^ |
| b | 19.26 mV |
| c | 9e-3 ms^-1^ |
| d | 22.03 mV |
| e | 1e-6 ms^-1^ |
| f | 16.26 mV |
| g | 29.79 mV |

**Supplementary Table 2.** The VDCC model equations and parameters from (Jaffe et al. 1994) and (Poirazi, Brannon, and Mel 2003).


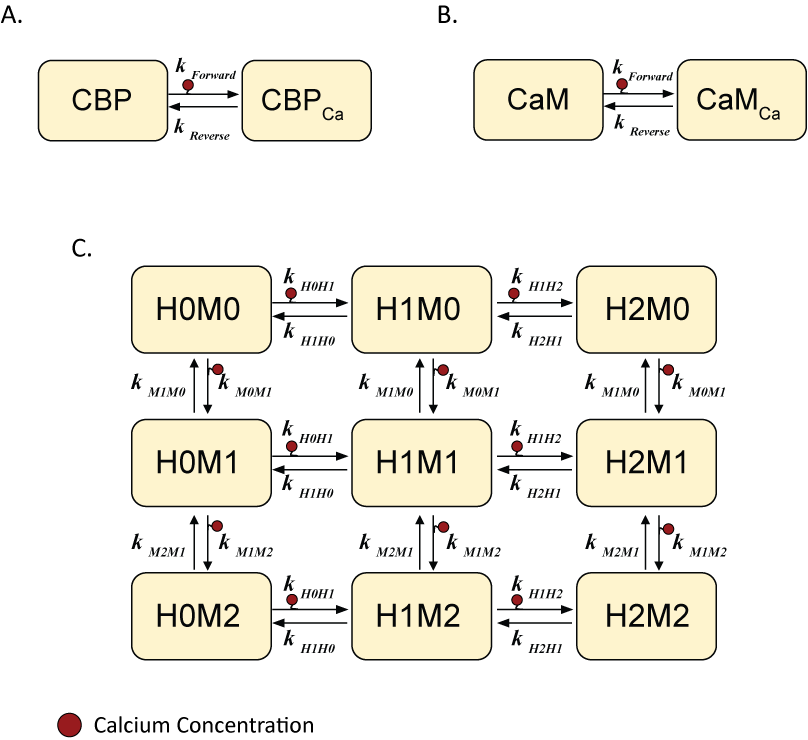


**Supplementary Figure 2.** Kinetic schema of the buffers present in the mechanistic model. Free calcium directly binds to the buffers, which shifts the buffer states from an unbound state to a bound state and changes the free calcium concentration. A. The kinetic schema of calcium binding proteins (Bartol et al. 2015; Naoki, Sakumura, and Ishii 2005). Parameter values are in Table 1. B. Kinetic schema of the calmodulin buffer (Zhabotinsky et al. 2006). Parameter values in Table 1. C. Kinetic schema of the calbindin buffer (Bartol et al. 2015). Parameters in Supplementary Table 3.

| **Parameter** | **Value** |
| --- | --- |
| $k_{M0M1}$ | 17.4.mM^-1^ ms^-1^ |
| $k_{M1M2}$ | 87 mM^-1^ ms^-1^ |
| $k_{M1M0}$ | 0.0358 ms^-1^ |
| $k_{M2M1}$ | 0.0716 ms^-1^ |
| $k_{H0H1}$ | 22. mM^-1^ ms^-1^ |
| $k_{H1H2}$ | 11 mM^-1^ ms^-1^ |
| $k_{H1H0}$ | 0.0026 ms^-1^ |
| $k_{H2H1}$ | 0.0052 ms^-1^ |

**Supplementary Table 3.** The calbindin rate constants from (Bartol et al. 2015).


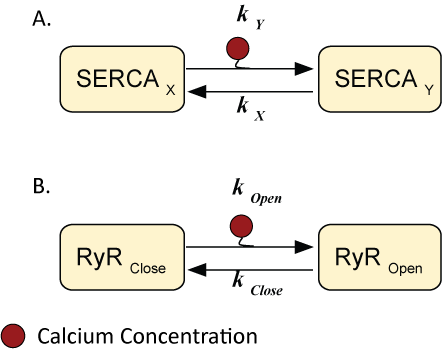


**Supplementary Figure 3.** Kinetic schema of the channels and pumps related to intracellular calcium. Free calcium interacts with the channels and pumps directly. A. The kinetic schema of SERCA pumps (Higgins et al. 2006). Equations and parameter values are in Supplementary Table 4. B. Kinetic schema of the Ryanodine receptor channel (Williams et al. 2011). Equations and parameter values in Supplementary Table 5.

| **Equations** | |
| --- | --- |
| $k_{x}=\frac{k_{2}\left[ Ca^{2+} \right]^{2}+k_{-4}K_{1}}{K_{1}+\left[ Ca^{2+} \right]^{2}}$ | |
| $k_{y}=\frac{k_{-2}{K_{3}\left[ Ca^{2+} \right]_{ER}}^{2}+k_{4}}{\gamma(1+{K_{3}\left[ Ca^{2+} \right]_{ER}}^{2})}$ | |
| **Parameter** | **Value** |
| $k_{2}$ | 600.ms^-1^ |
| $k_{-2}$ | 970 ms^-1^ |
| $k_{4}$ | 400 ms^-1^ |
| $k_{-4}$ | 1.2 ms^-1^ |
| $K_{1}$ | 8.4e-4 mmol/L |
| $K_{3}$ | 3.3333 mmol/L |
| $\gamma$ | 10 |

**Supplementary Table 4.** The SERCA model equations and parameters from (Higgins et al. 2006).

| **Equations** | |
| --- | --- |
| $k_{open}=\emptyset k^{+}\left[ Ca^{2+} \right]^{n}RyR_{close}$ | |
| $k_{close}=k^{-}RyR_{open}$ | |
| $\emptyset=\emptyset_{m}\left[ Ca^{2+} \right]_{ER}+\emptyset_{b}$ | |
| **Parameter** | **Value** |
| $\emptyset_{m}$ | 0.23.mM^-1^ |
| $\emptyset_{b}$ | 0.02 |
| $k^{+}$ | 47773 mM^-1^ms^-1^ |
| $k^{-}$ | 0.5 ms^-1^ |
| $n$ | 2.2 |

**Supplementary Table 5.** The Ryanodine receptor model equations and parameters from (Williams et al. 2011).

**
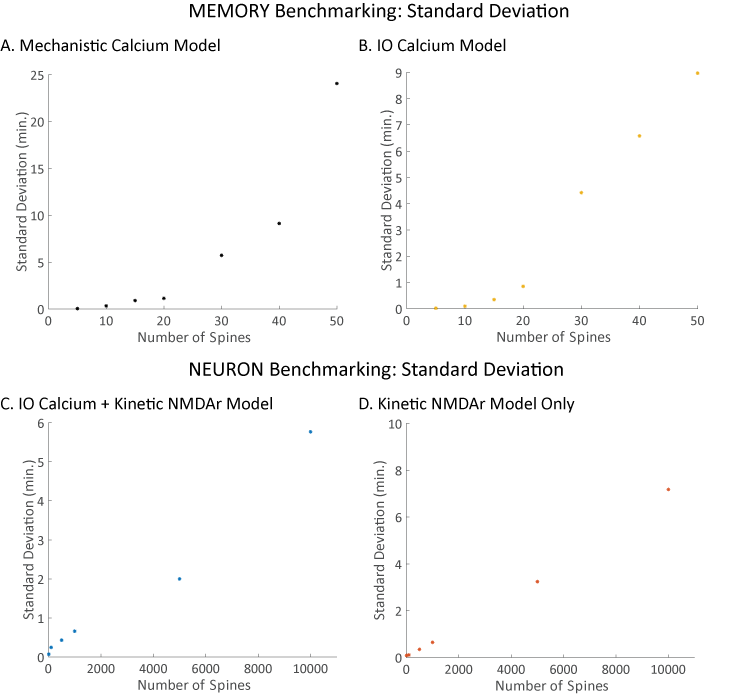
**

**Supplementary Figure 4.** The standard deviation values for the runtime benchmarking tests presented in the results section Figures 9 and 10. Each of the graphs represent the standard deviation values of: A. The mechanistic model in MEMORY; B. The input-output model in MEMORY; C. The input-output calcium model with the kinetic NMDAr model in NEURON; D. The kinetic NMDAr model in NEURON. The standard deviation of the runtimes is relatively minor compared to actual runtime values in all cases.

| 1st Order Coefficients | *c_1_ (1)* | 0.008800389723 |
| --- | --- | --- |
|  | *c****_1_*** *(2)* | -0.0005728031583 |
|  | *c****_1_*** *(3)* | -0.008696514657 |
|  | *c****_1_*** *(4)* | -0.0006856426334 |
|  | *c****_1_*** *(5)* | -3.34E-06 |
|  | *c****_1_*** *(6)* | -2.06E-05 |
|  | *c****_1_*** *(7)* | 2.89E-05 |
|  | *c****_1_*** *(8)* | -1.91E-05 |
| 2nd Order Coefficients | *c****_2s_*** *(1,1)* | 0.0002334077521 |
|  | *c****_2s_*** *(1,2)* | 0.00108240597 |
|  | *c****_2s_*** *(1,3)* | -7.84E-05 |
|  | *c****_2s_*** *(1,4)* | 0.0003611022086 |
|  | *c****_2r_*** *(1,5)* | -0.001140775718 |
|  | *c****_2r_*** *(1,6)* | -0.0005952583753 |
|  | *c****_2r_*** *(1,7)* | -0.002228671784 |
|  | *c****_2r_*** *(1,8)* | 7.13E-05 |
|  | *c****_2s_*** *(2,2)* | 0.002162946784 |
|  | *c****_2s_*** *(2,3)* | 0.0001678369414 |
|  | *c****_2s_*** *(2,4)* | 0.001120695263 |
|  | *c****_2r_*** *(2,5)* | -7.79E-05 |
|  | *c****_2r_*** *(2,6)* | -0.001118081226 |
|  | *c****_2r_*** *(2,7)* | -7.91E-05 |
|  | *c****_2r_*** *(2,8)* | 1.50E-06 |
|  | *c****_2s_*** *(3,3)* | 0.001171468345 |
|  | *c****_2s_*** *(3,4)* | -8.85E-05 |
|  | *c****_2r_*** *(3,5)* | -0.001169106422 |
|  | *c****_2r_*** *(3,6)* | -7.62E-05 |
|  | *c****_2r_*** *(3,7)* | 2.43E-06 |
|  | *c****_2r_*** *(3,8)* | 3.26E-07 |
|  | *c****_2s_*** *(4,4)* | -0.0005300240024 |
|  | *c****_2r_*** *(4,5)* | 5.00E-05 |
|  | *c****_2r_*** *(4,6)* | 0.0005293753636 |
|  | *c****_2r_*** *(4,7)* | 1.81E-05 |
|  | *c****_2r_*** *(4,8)* | 1.10E-06 |
|  | *c****_2s_*** *(5,5)* | -7.71E-08 |
|  | *c****_2s_*** *(5,6)* | -3.62E-06 |
|  | *c****_2s_*** *(5,7)* | -0.0006897547887 |
|  | *c****_2s_*** *(5,8)* | 4.04E-05 |
|  | *c****_2s_*** *(6,6)* | 0.0006895191522 |
|  | *c****_2s_*** *(6,7)* | 6.36E-05 |
|  | *c****_2s_*** *(6,8)* | -2.47E-06 |
|  | *c****_2s_*** *(7,7)* | 4.86E-07 |
|  | *c****_2s_*** *(7,8)* | 4.43E-06 |
|  | *c****_2s_*** *(8,8)* | -1.01E-06 |
| 3rd Order Coefficients | *c****_3s_*** *(1,1,1)* | 0.00101033653 |
|  | *c****_3s_*** *(1,1,2)* | -0.002010932034 |
|  | *c****_3s_*** *(1,1,3)* | 0.0003095523277 |
|  | *c****_3s_*** *(1,1,4)* | -1.23E-05 |
|  | *c****_3r_*** *(1,1,5)* | -0.00145206025 |
|  | *c****_3r_*** *(1,1,6)* | 0.003775342189 |
|  | *c****_3r_*** *(1,1,7)* | -0.0002998500677 |
|  | *c****_3r_*** *(1,1,8)* | -0.000140108151 |
|  | *c****_3s_*** *(1,2,2)* | -0.001763371673 |
|  | *c****_3s_*** *(1,2,3)* | 0.0005818571955 |
|  | *c****_3s_*** *(1,2,4)* | 0.00489291987 |
|  | *c****_3r_*** *(1,2,5)* | -0.0005060899415 |
|  | *c****_3r_*** *(1,2,6)* | 8.87E-06 |
|  | *c****_3r_*** *(1,2,7)* | -0.009981584889 |
|  | *c****_3r_*** *(1,2,8)* | 0.0005213859892 |
|  | *c****_3s_*** *(1,3,3)* | 0.005089582413 |
|  | *c****_3s_*** *(1,3,4)* | -0.0006989076058 |
|  | *c****_3r_*** *(1,3,5)* | 4.49E-05 |
|  | *c****_3r_*** *(1,3,6)* | 0.0007048530348 |
|  | *c****_3r_*** *(1,3,7)* | 2.47E-05 |
|  | *c****_3r_*** *(1,3,8)* | 0.002640291568 |
|  | *c****_3s_*** *(1,4,4)* | -0.0003504451115 |
|  | *c****_3r_*** *(1,4,5)* | 1.18E-05 |
|  | *c****_3r_*** *(1,4,6)* | -0.005296178713 |
|  | *c****_3r_*** *(1,4,7)* | 0.0003516732956 |
|  | *c****_3r_*** *(1,4,8)* | 0.002655906336 |
|  | *c****_3r_*** *(1,5,5)* | -0.0004383885842 |
|  | *c****_3r_*** *(1,5,6)* | 2.85E-05 |
|  | *c****_3r_*** *(1,5,7)* | 0.0004393474112 |
|  | *c****_3r_*** *(1,5,8)* | 1.84E-05 |
|  | *c****_3r_*** *(1,6,6)* | 0.000341853196 |
|  | *c****_3r_*** *(1,6,7)* | -2.52E-05 |
|  | *c****_3r_*** *(1,6,8)* | -0.0003416574756 |
|  | *c****_3r_*** *(1,7,7)* | -2.39E-05 |
|  | *c****_3r_*** *(1,7,8)* | -1.20E-06 |
|  | *c****_3r_*** *(1,8,8)* | 0.009165395727 |
|  | *c****_3s_*** *(2,2,2)* | -0.001392576948 |
|  | *c****_3s_*** *(2,2,3)* | 5.31E-05 |
|  | *c****_3s_*** *(2,2,4)* | -0.018345539 |
|  | *c****_3r_*** *(2,2,5)* | 0.001394102698 |
|  | *c****_3r_*** *(2,2,6)* | 0.009180067762 |
|  | *c****_3r_*** *(2,2,7)* | -0.001284747206 |
|  | *c****_3r_*** *(2,2,8)* | 9.70E-05 |
|  | *c****_3s_*** *(2,3,3)* | 0.001285257947 |
|  | *c****_3s_*** *(2,3,4)* | 4.53E-05 |
|  | *c****_3r_*** *(2,3,5)* | 0.0003401173163 |
|  | *c****_3r_*** *(2,3,6)* | -2.47E-05 |
|  | *c****_3r_*** *(2,3,7)* | -0.0003401861924 |
|  | *c****_3r_*** *(2,3,8)* | -2.38E-05 |
|  | *c****_3s_*** *(2,4,4)* | -2.08E-06 |
|  | *c****_3r_*** *(2,4,5)* | 6.42E-05 |
|  | *c****_3r_*** *(2,4,6)* | -4.46E-06 |
|  | *c****_3r_*** *(2,4,7)* | -6.48E-05 |
|  | *c****_3r_*** *(2,4,8)* | -4.52E-06 |
|  | *c****_3r_*** *(2,5,5)* | -1.78E-06 |
|  | *c****_3r_*** *(2,5,6)* | -6.37E-07 |
|  | *c****_3r_*** *(2,5,7)* | -0.007661851468 |
|  | *c****_3r_*** *(2,5,8)* | 0.001092090514 |
|  | *c****_3r_*** *(2,6,6)* | -3.94E-05 |
|  | *c****_3r_*** *(2,6,7)* | 0.0153487621 |
|  | *c****_3r_*** *(2,6,8)* | -0.00109454611 |
|  | *c****_3r_*** *(2,7,7)* | -0.007686870628 |
|  | *c****_3r_*** *(2,7,8)* | 0.001132691024 |
|  | *c****_3r_*** *(2,8,8)* | -7.94E-05 |
|  | *c****_3s_*** *(3,3,3)* | -0.001133695993 |
|  | *c****_3s_*** *(3,3,4)* | -4.26E-05 |
|  | *c****_3r_*** *(3,3,5)* | -0.0004986861998 |
|  | *c****_3r_*** *(3,3,6)* | 3.66E-05 |
|  | *c****_3r_*** *(3,3,7)* | 0.0004984153138 |
|  | *c****_3r_*** *(3,3,8)* | 3.49E-05 |
|  | *c****_3s_*** *(3,4,4)* | 3.45E-06 |
|  | *c****_3r_*** *(3,4,5)* | -0.0001572892445 |
|  | *c****_3r_*** *(3,4,6)* | 1.08E-05 |
|  | *c****_3r_*** *(3,4,7)* | 0.0001580578656 |
|  | *c****_3r_*** *(3,4,8)* | 1.13E-05 |
|  | *c****_3r_*** *(3,5,5)* | 4.59E-06 |
|  | *c****_3r_*** *(3,5,6)* | 2.46E-06 |
|  | *c****_3r_*** *(3,5,7)* | 0.0001436643616 |
|  | *c****_3r_*** *(3,5,8)* | -1.02E-05 |
|  | *c****_3r_*** *(3,6,6)* | -0.0001438402566 |
|  | *c****_3r_*** *(3,6,7)* | -1.03E-05 |
|  | *c****_3r_*** *(3,6,8)* | -3.51E-06 |
|  | *c****_3r_*** *(3,7,7)* | -2.94E-06 |
|  | *c****_3r_*** *(3,7,8)* | 1.35E-06 |
|  | *c****_3r_*** *(3,8,8)* | 0.0008844405737 |
|  | *c****_3s_*** *(4,4,4)* | -0.0001106519827 |
|  | *c****_3r_*** *(4,4,5)* | 3.59E-06 |
|  | *c****_3r_*** *(4,4,6)* | -0.0017862926 |
|  | *c****_3r_*** *(4,4,7)* | 0.0001121804722 |
|  | *c****_3r_*** *(4,4,8)* | 0.0009018099373 |
|  | *c****_3r_*** *(4,5,5)* | -0.0001277678698 |
|  | *c****_3r_*** *(4,5,6)* | 7.44E-06 |
|  | *c****_3r_*** *(4,5,7)* | 0.0001286986701 |
|  | *c****_3r_*** *(4,5,8)* | 4.92E-06 |
|  | *c****_3r_*** *(4,6,6)* | 0.0001291620016 |
|  | *c****_3r_*** *(4,6,7)* | -9.40E-06 |
|  | *c****_3r_*** *(4,6,8)* | -0.00012908639 |
|  | *c****_3r_*** *(4,7,7)* | -9.24E-06 |
|  | *c****_3r_*** *(4,7,8)* | -1.51E-06 |
|  | *c****_3r_*** *(4,8,8)* | -3.93E-06 |
|  | *c****_3s_*** *(5,5,5)* | 6.74E-07 |
|  | *c****_3s_*** *(5,5,6)* | 3.62E-06 |
|  | *c****_3s_*** *(5,5,7)* | 5.79E-08 |
|  | *c****_3s_*** *(5,5,8)* | -1.99E-06 |
|  | *c****_3s_*** *(5,6,6)* | -1.09E-06 |
|  | *c****_3s_*** *(5,6,7)* | -6.16E-05 |
|  | *c****_3s_*** *(5,6,8)* | 4.06E-06 |
|  | *c****_3s_*** *(5,7,7)* | 6.18E-05 |
|  | *c****_3s_*** *(5,7,8)* | 4.83E-06 |
|  | *c****_3s_*** *(5,8,8)* | 3.01E-06 |
|  | *c****_3s_*** *(6,6,6)* | 2.46E-06 |
|  | *c****_3s_*** *(6,6,7)* | -1.69E-06 |
|  | *c****_3s_*** *(6,6,8)* | 1.50E-05 |
|  | *c****_3s_*** *(6,7,7)* | -9.25E-07 |
|  | *c****_3s_*** *(6,7,8)* | -1.50E-05 |
|  | *c****_3s_*** *(6,8,8)* | -1.28E-06 |
|  | *c****_3s_*** *(7,7,7)* | -5.73E-07 |
|  | *c****_3s_*** *(7,7,8)* | -4.72E-07 |
|  | *c****_3s_*** *(7,8,8)* | 6.48E-07 |
|  | *c****_3s_*** *(8,8,8)* | -8.27E-08 |

**Supplementary Table 6.** Coefficient values estimated for the Input-Output calcium model. In the second column, the coefficient parameters weight the various basis functions that are cross multiplied with each other in the multi-input single output model. Numbers 1-4 correlate with the basis functions convolved with NMDA receptor conductance (gTotalNMDA) input, and numbers 5-8 correlate with the basis functions convolved with the postsynaptic potential (V) input.
